# Fighting cytokine storm and immunomodulatory deficiency: By using natural products therapy up to now

**DOI:** 10.3389/fphar.2023.1111329

**Published:** 2023-04-12

**Authors:** Mona A. Mohammed

**Affiliations:** Medicinal and Aromatic Plants Research Department, Pharmaceutical and Drug Industries Research Institute, National Research Centre, Giza, Egypt

**Keywords:** cytokine storm, immunomodulatory, natural products, SARS-CoV-2, clinical trials drugs

## Abstract

A novel coronavirus strain (COVID-19) caused severe illness and mortality worldwide from 31 December 2019 to 21 March 2023. As of this writing, 761,071,826 million cases have been diagnosed worldwide, with 6,879,677 million deaths accorded by WHO organization and has spread to 228 countries. The number of deaths is closely connected to the growth of innate immune cells in the lungs, mainly macrophages, which generate inflammatory cytokines (especially IL-6 and IL-1β) that induce “cytokine storm syndrome” (CSS), multi-organ failure, and death. We focus on promising natural products and their biologically active chemical constituents as potential phytopharmaceuticals that target virus-induced pro-inflammatory cytokines. Successful therapy for this condition is currently rare, and the introduction of an effective vaccine might take months. Blocking viral entrance and replication and regulating humoral and cellular immunity in the uninfected population are the most often employed treatment approaches for viral infections. Unfortunately, no presently FDA-approved medicine can prevent or reduce SARS-CoV-2 access and reproduction. Until now, the most important element in disease severity has been the host’s immune response activation or suppression. Several medicines have been adapted for COVID-19 patients, including arbidol, favipiravir, ribavirin, lopinavir, ritonavir, hydroxychloroquine, chloroquine, dexamethasone, and anti-inflammatory pharmaceutical drugs, such as tocilizumab, glucocorticoids, anakinra (IL-1β cytokine inhibition), and siltuximab (IL-6 cytokine inhibition). However, these synthetic medications and therapies have several side effects, including heart failure, permanent retinal damage in the case of hydroxyl-chloroquine, and liver destruction in the case of remdesivir. This review summarizes four strategies for fighting cytokine storms and immunomodulatory deficiency induced by COVID-19 using natural product therapy as a potential therapeutic measure to control cytokine storms.

## Graphical Abstract

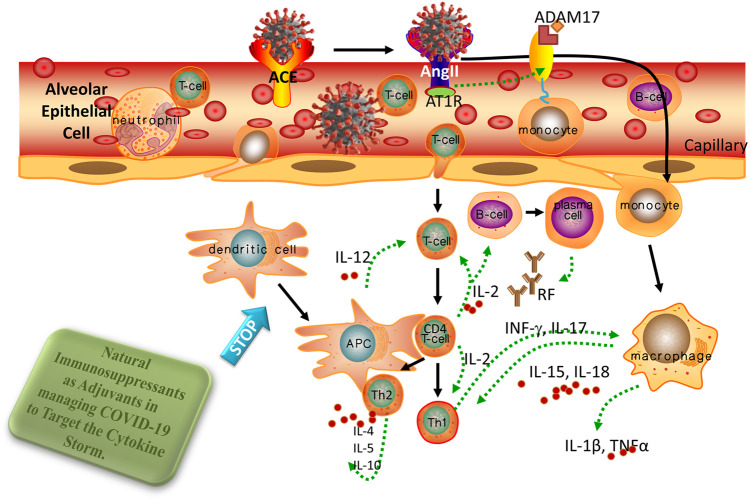

## Highlights

⁃ The immunopathogenesis of the cytokine storms seen in severe and acute respiratory-CoV-2 infection is briefly described in this review article.
⁃ Several natural compounds as immunosuppressants that can be employed as adjuvants in the treatment of COVID-19 diseases are discussed.

## 1 Introduction

A virus can activate the immune system (B-cells, T-cells, macrophages, neutrophil cells, dendritic cells, and monocytes cells) and resident tissue cells, producing massive amounts of pro-inflammatory cytokines (Chugh et al., 2023). During a flu virus infection, innate immune responses are triggered by interferon (IFN)-stimulated gene regulation cascade amplification events, and IFN is mainly produced by macrophages, monocytes, and dendritic cells.

### 1.1 Global crisis

IAVG spearheaded the effort to achieve 70% coverage with COVID-19 vaccination rates in all nations as a global goal (Knoll and Wonodi, 2021). COVAX’s initial strategy was to attain 3% vaccination coverage, followed by 20% coverage with COVAX-secured doses by the end of 2021 (World Health Organization, 2020). In October 2021, the World Health Organization (WHO) unveiled strategies to reach global coverage for the COVID-19 vaccine by the middle of 2022. These goals were to expand globally (World Health Organization, 2020). By the end of 2021, the revised global aim was 40% total population coverage, with 70% total population coverage by mid-2022. These data, however, came from all countries’ sources of supply, not only COVAX. Conversely, COVAX would make every effort to accomplish this coverage level in a fair and equitable manner (Knoll and Wonodi, 2021).

**FIGURE 1:**
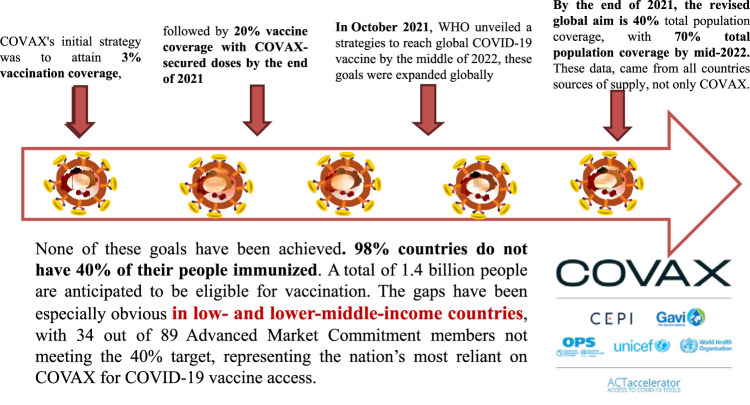
Flowcharts of “WHO unveiled strategies to reach global COVID-19 vaccine”. IAVG spearheaded the effort to achieve 70 percent coverage with COVID-19 vaccination rates in all nations as a global goals need. (Adapted from Knoll and Wonodi, 2021).

None of these goals have been achieved. In total, 98 countries do not have 40% of their people immunized. A total of 1.4 billion people are anticipated to be eligible for vaccination (Peacocke et al., 2021), many of whom are in the highest risk groups for death and serious illness. The gaps have been especially obvious in low- and lower-middle-income countries (LICs and LMICs), with 34 of 89 advanced market commitment (AMC) members not meeting the 40% target, representing the nations most reliant on COVAX for COVID-19 vaccine access (Figure 1) (Zhang et al., 2020a).

### 1.2 COVID-19 pathogenesis

**FIGURE 2:**
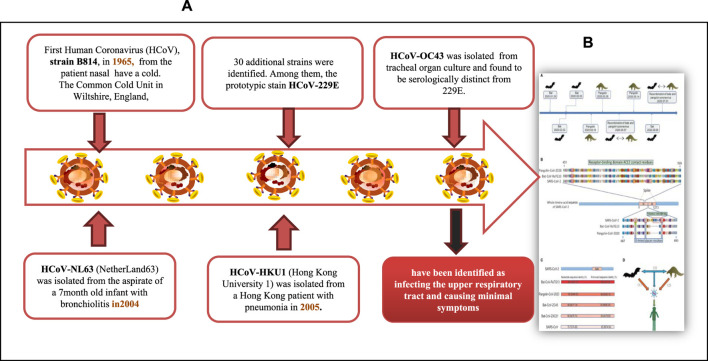
**(A)** COVID-19 Pathogenesis and **(B)** A member of a wide family of encapsulated, non-segmented, positives (+) RNA viruses’ sense single-stranded that can be found in dogs, camels, bats, and cats. (Adapted from Chen et al., 2021).

Coronavirus is an encapsulated, non-segmented, and positive (+) RNA virus with a single-stranded sense found in animals, such as dogs, camels, bats, and cats. Because their structure resembles a crown or corona, these viruses are called coronaviruses. Coronaviruses infect humans and animals. For humans, four coronavirus strains (NL63, HKU1, OC43, and 229E) have been identified as infecting the upper respiratory tract and causing minimal symptoms (Fehr and Perlman, 2015). There are three human coronavirus strains (2019-nCoV-2, also called SARS-CoV-2, SARS-CoV, and MERS-CoV) have been identified as infecting the Lower Respiratory Tract, causing pneumonia and Death (Figure 2A). The genetic sequence of SARS-CoV-2, also known as the severe acute respiratory syndrome coronavirus, is 79% similar to that of SARS-CoV and 98% similar to the RaTG13 coronaviruses found in bats chrysanthemum (Figure 2B) (Zhou et al., 2020; Chen et al., 2021). Furthermore, SARS immunopathogenesis, caused by CoV-2, which causes airway damage, is quite similar to that of SARS-CoV. ARDS develops in extreme symptoms of SARS-CoV-2 infection, resulting in respiratory organ failure, the major cause of death. 3) Hong Kong University 1 (HKU1) isolated the Middle East respiratory syndrome coronavirus (MERS-CoV) in 2005 from a pneumonia patient in Hong Kong (Zhang et al., 2020a). Furthermore, the rise in immunological responses to viral infection causes increased quantities of inflammatory cytokines to be released by innate immune cells, resulting in the development of “cytokine storm syndrome” (CSS) (Zhang et al., 2020a). This illness causes uncontrollable inflammation, which causes numerous organ failures and eventually death. As a result of these observations, we can deduce that viral infectious diseases are not the main cause of airway damage. However, the main immune response is also important in disease progression. Additionally, older age and the presence of co-morbidities have been connected to the severity of the condition (Figure 2) (Guan et al., 2020).

### 1.3 With the problem of the COVID-19 pandemic

Due to the lack of a specialized antiviral to end the infection and a proper vaccination to dissolve it, the COVID-19 epidemic has resulted in a major global health crisis and social unrest. Some people infected with severe acute respiratory CoV-2 have a well-coordinated immune response and recover. In contrast, others have a faulty immune response and do not recover, which can lead to catastrophic consequences. The secretion of enormous amounts of active mediators and inflammatory substances during a cytokine storm not only prevents the virus from spreading further in the body but also causes secondary tissue damage (Tang et al., 2020). A large cytokine and chemokine release, referred to as a “cytokine storm,” has been discovered in patients with a malfunctioning immune response.

Because the cytokine storm is the main cause of the high death rate in COVID-19 patients, it has become a key therapeutic target for reversing the disease’s progression in severely and critically ill individuals.

• Although specific causes of cytokine storms in COVID-19 have not been fully elucidated, 1) hyper-incited activated innate immune responses and 2) ACE-2 dysregulation have been proposed.
• Imagination and expression: To combat cytokine storms, immunoregulatory medications, including 1) cytokine inhibitors, 2) all corticosteroids, 3) blood purifying therapy, and 4) mesenchymal stem cell treatments, have been used (Tang et al., 2020).

Since December 2019, the pandemic, which is caused by a novel coronavirus called 2019-CoV-2 (also SARS-CoV-2) and was originally diagnosed in Wuhan, China, has prompted acute and grave global concern (Bogoch et al., 2020; Wang et al., 2020b). The WHO reportedly proclaimed COVID-19 a global epidemic on 11 March 2020, posing a serious threat to people’s health and societal stability. Severe acute respiratory infection symptoms (SARS) appeared early in the stages of COVID-19, and abrupt respiratory distress syndrome (ARDS), acute respiratory failure, and other significant consequences plagued many patients.

Much evidence indicates immunologically pathogenic changes as follows:

1) Reduced lymphocytes.
2) In COVID-19 patients, especially those severely sick, elevated cytokines are major drivers of disease progression and death.

## 2 Cytokine storm

The term “cytokine storm” was used to characterize the excessive immune responses that can be generated by a range of events, including virus infection, autoimmune illness, and immunotherapies. It was first used to describe RA and GVHD (Shimabukuro-Vornhagen et al., 2018).

### 2.1 Viral infection causes a cytokine storm

On 21 March 2023, there are 761,071,826 confirmed COVID-19 cases worldwide, including 6,879,677 deaths reported to the WHO by national authorities, and has spread to 228 countries (Chugh et al., 2023). Many studies indicate that in addition to the use of antiviral medicines to treat COVID-19, the reduction of the cytokine storm might be an effective therapeutic strategy for successfully combating the disease (Rahmati and Moosavi, 2020).

The virus can activate the immune cells system (e.g., B cells, T cells, macrophages, neutrophil cells, dendritic cells, and monocytes cells) and resident tissue cells, producing massive amounts of pro-inflammatory cytokines (Krischuns et al., 2018). During a flu virus infection, innate immune responses are triggered by IFN-stimulated gene regulation cascade amplification events, and IFN is primarily created by macrophages, monocytes, and dendritic cells (Klinkhammer et al., 2018).

**FIGURE 3:**
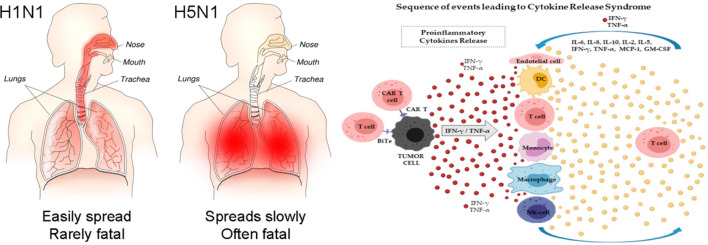
(Left) Severe viral infection, a cytokine storm develops in the lungs (Author: Tim Vickers, Source: https://upload.wikimedia.org/wikipedia/commons/b/b0/H1N1_versus_H5N1_pathology.png. (Right) The pathophysiology of cytokine storm (Adapted from Cosenza et al., 2021). (1)Viruses infect lung epithelial cells and alveolar macrophages, resulting in the production of cytokines /chemokines are produced by viruses infection (fundamentally have interferons). (2) Cytokines & chemokines that activate a macrophage and virally destroy dendritic cells initiate a cytokine storm, resulting in a more widespread immune response. (3) Chemokines released in the bloodstream attract more inflammatory cells to the irritation site, and these cells then release more inflammatory chemokines & cytokines, amplifying the “cytokine storms”.

In H5N1 (influenza virus infection), the serum concentration levels of IFN-induced protein-10 (IP-10), interleukin-8 (IL-8), macrophage inflammatory protein-1(MIP-1), monocyte chemoattractant protein-1 (MCP-1), monokine stimulated by IFN-γ (MIG), and CXC chemokine ligand-9 (CXCL-9) were anomalously elevated, whereas IL-17, IL-9, IL-8, IL-6, IL-15, tumor necrosis factor-alpha (TNF-α), and IL-10 were abnormally elevated in H1N1 and influenza virus infection (Thomas et al., 2017). Serum concentrations of pro-inflammatory factors [IFN-γ, IL-1β, IL-12, IL-6, IL-8, IL-18, IP-10, MCP-1, CC-chemokine ligand-2 (CCL-2), and CXCL-10] are positively connected with pulmonary fibrosis and significant lung tissue destruction in SARS-coronavirus patients, according to the latest research (Song et al., 2020; Rabaan et al., 2021) (Figure 3E). However, in MERS-coronavirus severe patients, serum levels of pro-inflammatory cytokines IL-6, IL-15, IL-17, IFN-γ, TNF-α, and chemokines IL-8, CXCL-10, and CCL-5 appeared significantly elevated (Mahallawi et al., 2018). The relevance of molecules (e.g., IL-6, IFN-γ, IL-1β, IL-8, IL-10, and TNF-α) increases in virally mediated cytokine storms (Chousterman et al., 2017). As a result, the cytokine storm has been identified as one of the leading causes of death in patients infected with SARS-CoV, MERS-CoV, or other influenza viruses (Dawood et al., 2012). Similarly, in COVID-19, cytokine storm is a typical hallmark of severe cases, and increased serum IL-6 and CRP levels are related to respiratory distress, MOF, ARDS, and poor clinical outcomes (Gómez-Pastora et al., 2020). Another research group recorded 70 reported-long COVID-19 outcomes in unvaccinated patients infected with SARS-CoV-2 matched to uninfected people, adjusted for age and sex and stratified by SARS-CoV-2 variants, and risk in patients with a breakthrough SARS-CoV-2 infection compared with unvaccinated infected controls. Risks were compared using hazard ratios and risk differences per 10,000 patients measured during the early (30–180 days) and late (180–360 days) time periods after infection. The result found that the COVID-19 infection was significantly associated with increased risks in early and late periods for anosmia and dysgeusia and a risk difference with a 95% confidence interval (Mizrahi et al., 2023).

### 2.2 Cytokine release syndrome (CRS)

A severe, life-threatening illness that can emerge as a result of an infection autoimmune disease is referred to as a cytokine storm or hyper-cytokinemia. Patients infected with SARS-coronavirus and MERS-coronavirus, as well as leukemia patients receiving engineered T-cell therapies, have previously experienced a type of systemic inflammatory syndrome produced by cytokine storms (Fehr et al., 2017; Shimabukuro-Vornhagen et al., 2018). Fever, tiredness, headache, rash, arthralgia, and myalgia are symptoms of moderate cases. High temperature, headache, exhaustion, diffuse intravascular coagulation (DIC), shock, multiple organ failure (MOF), and even death are common symptoms in cases of severe symptoms (Shimabukuro-Vornhagen et al., 2018). Cytopenia, increased creatinine and liver functions, elevated concentrations of C-reactive protein (CRP), and abnormal coagulation parameters are common lab findings (Shimabukuro-Vornhagen et al., 2018). Lee and the lab team described a modified scoring system again for the severity of cytokine release syndrome (CRS), which categorized mild, moderate, severe, and life-threatening symptoms and even death, disregarding the initiating cause (Lee et al., 2014). Clinical decisions in CRS are similarly guided by this grading system. Immune suppressants should be utilized in all sick cases with grade 3 or 4 CRS and, therefore, should be started earlier in patients with substantial comorbidity or the elderly (Shimabukuro-Vornhagen et al., 2018) In Figure 3A, type II pneumocytes in airways and alveoli are first infected by SARS-CoV-2, which causes alveolar macrophages to become activated, cytokines to be produced, and further inflammatory infiltration. The SARS-CoV-2 viral lifecycle is depicted in simplified form in Figure 3B, with the creation of non-structural proteins (NSPs) that block the early IFN response in infected cells, which normally limits viral multiplication. This virulence pathway could indirectly cause an unbalanced inflammatory response. S-CSS typically affects adults and various organ systems, most notably the lymphoid, lung, heart, liver, gastrointestinal, and renal systems (Figure 3C). Multiorgan dysfunction and lung damage may feed off of each other negatively. The majority of children who develop MIS-C (Figure 3D) present with distinctive characteristics, such as pronounced cardiac and gastrointestinal involvement and less frequent or milder involvement of other systems (Cabler et al., 2020).

### 2.3 The pathophysiology and key of cytokine storms to determine COVID-19

Proliferating lung epithelial cells, which are the initial vectors for virus infection, can attack other cells, including pulmonary macrophages (Liu et al., 2016). When infected cells are destroyed by apoptosis or necrosis, inflammation reactions are initiated. The acute inflammatory process is the organism’s first response to damaging triggers. It is characterized by increased blood flow, which allows serum and leukocytes to reach extravascular areas of damage, the elevation of heating rate, and discomfort. The activation of pro-inflammatory cytokines or chemokines is also a sign of an acute inflammatory process response (Thomas et al., 2017). Several pro-inflammatory cytokines or chemokines can cause inflammatory cells to be recruited (Thomas et al., 2017). Overexpression of inflammation and antiviral and apoptosis genes occurs in conjunction with extensive immune cell infiltration and tissue injury, followed by the initiation of regeneration processes and damage repair. In some cases, this reparative technique can entirely recover function. More substantial pathological alterations, including multiorgan destruction, hyaline membrane development, fibrin exudates, and fibrotic healing, are evident in severe inflammation coupled with cytokine storms. When a virus infects a cell, it produces damage-associated molecular patterns (DAMPs) and pathogen-associated molecular patterns (PAMPs), which could stimulate antiviral reaction responses through neighboring cells and attract innate and adaptive immune cells such as macrophages, natural killer (NK) cells, and gamma delta T (γδT) cells. These symptoms indicate extensive capillary destruction, immunopathologic damage, and chronic organ dysfunction. Furthermore, strong inflammatory cytokines/chemokines could leak into the bloodstream and cause systemic cytokine storms, which cause multi-organ failure. Whenever the virus’s PAMP is identified by the innate immune cells’ system of pattern recognition receptors (PRRs), the inflammatory process begins. After that, the downstream signaling cascades of PRRs are initiated, and specific pro-inflammatory cytokines are produced (Figure 3F).

**FIGURE 4:**
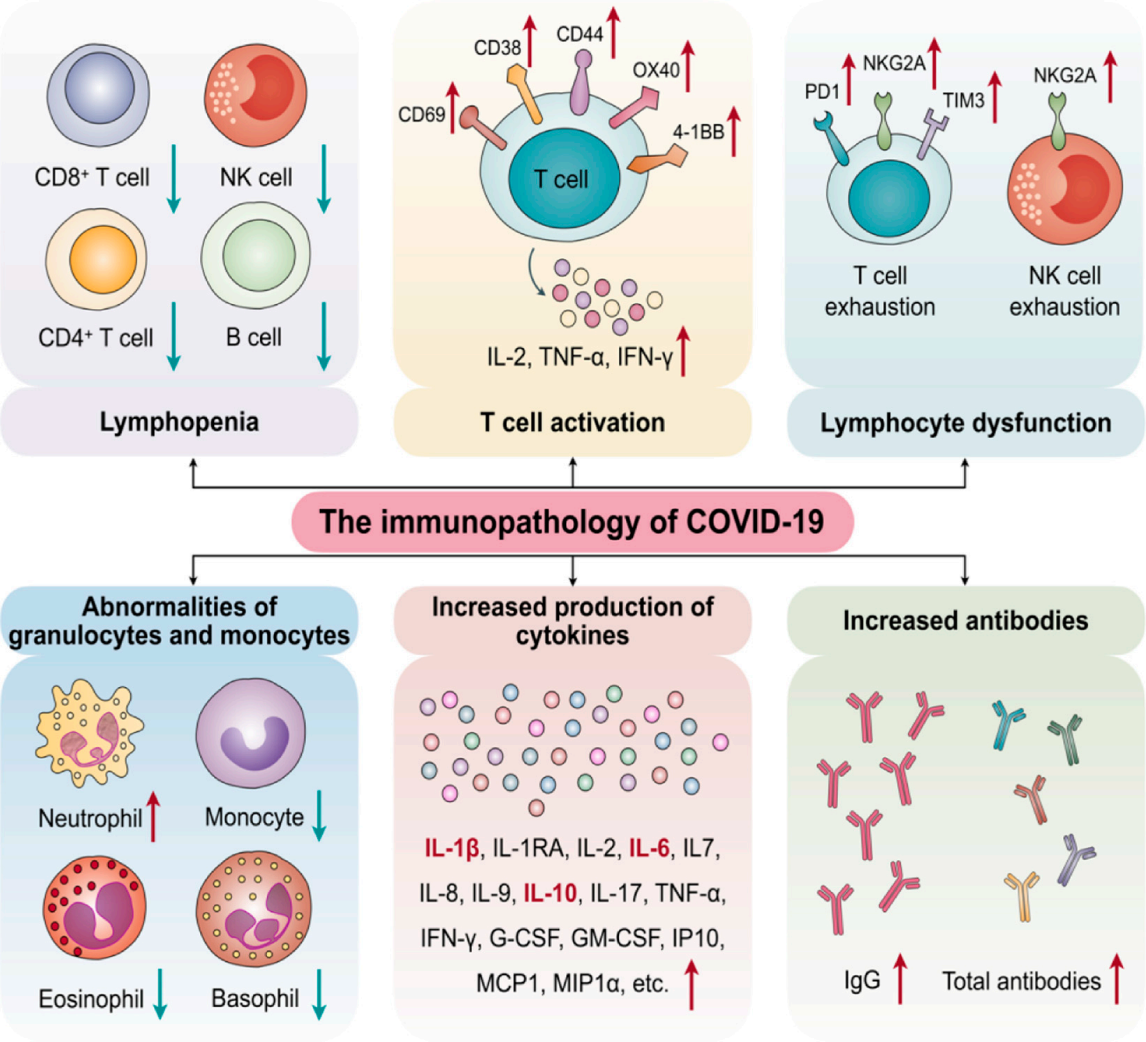
The key of Cytokine Storms to determine SARS/COVID-19 in immunopathology (Adapted from Yang et al., 2020a).

Patients in the intensive care unit (ICU) had significantly greater concentration levels of plasma-inflammatory cytokines such as IL-2, IL-10, IL-7, granulocyte-colony-stimulating factor (G-CSF), MCP, IFN-γ, and TNF-α than non-ICU patients (Huang et al., 2020), demonstrating a link between the cytokine storms and the severity of the disease. In COVID-19, which differed from SARS-CoV, these cytokines predicted not only Th1-cell response but also Th2-cell responses. CD4^+^ T-cells were stimulated and differentiated into Th1 cells following infection with SARS-CoV-2, secreting plasma-pro-inflammatory cytokines including IL-6, IFN-γ, and granulocyte–macrophage-colony-stimulating factor (GM-CSF) (Huang et al., 2020). GM-CSF may stimulate mononuclear cells, causing them to release more IL-6 and other pro-inflammatory cytokines, resulting in massive cytokine storms. As a result, the cytokine storm in SARS/COVID-19 may be a key mediated by IL-6 and GM-CSF secreted by mononuclear and T lymphocyte cells. Furthermore, the activation of monocytes could indicate that the cytokine storms in SARS/COVID-19 are linked to the breakdown of the innate-adaptive immune balance. Recent researchers also found that although the level of IL-6 in chronic COVID-19 patients was much higher than that in mild and moderate cases, the levels of CD4^+^ T cells, CD8^+^ T cells, and NK cells were lower, indicating immunosuppression (Wan et al., 2020). Although the number of CD4^+^ and CD8^+^ T cells in the peripheral blood was reduced, the number of Th17 cells was elevated, and CD8^+^ T cells were highly cytotoxic, suggesting that a cytokine storm may accelerate tissue destruction (Wan et al., 2020). Meanwhile, during the cytokine storms, a T lymphocyte cell becomes too activated, according to Wan et al. (2020), in Chongqing Hospital, China (Figure 4).

### 2.4 Mechanisms of cytokine storm helping in COVID-19

#### 2.4.1 Hyper-activated innate immune responses

**FIGURE 5:**
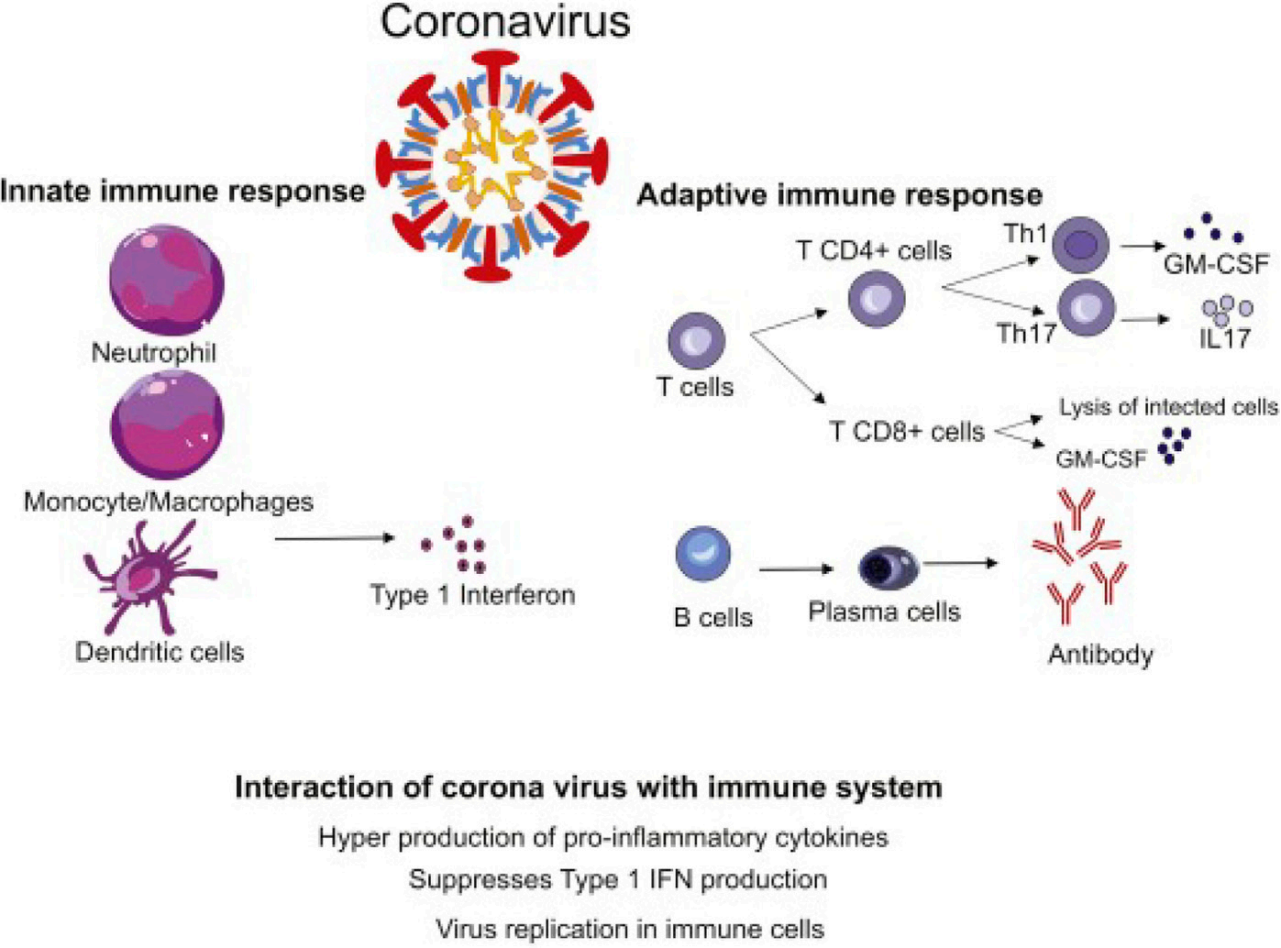
The immunological responses against COVID-9-coronavirus (CoV) disease, in both cases innate and adaptive immune responses. The secretion of numerous pro-inflammatory cytokines by neutrophils cells, monocytes cells, macrophages, and dendritic cells is referred to as a “cytokine storm.” Pulmonary immunopathology is the result of this process. Th1 cells and Th17 cells and CD+ T cells may be stimulated, exacerbating lung damage. By lysis of tumor cells, the cytotoxic T-lymphocyte (CTL) contributes to antiviral therapy. Antibodies specific to viruses are produced by B cells, and viruses are neutralized by them. (Adapted from Hosseini et al., 2020).

Adaptive and innate immune responses connect and then collaborate in close proximity to create immunological defense during the antiviral immune response process (Hosseini et al., 2020). There is a time limit for adaptive immune responses, which usually gets initiated 4–7 days after infection. Unlike adaptive immune responses, innate immune responses occur immediately after infection and are fully involved in virus clearance. However, innate immunity is relatively weak in virus clearance, and adaptive immunity is the key factor in the complete elimination of the virus (Hosseini et al., 2020). If the body does not generate effective adaptive antiviral responses in time to clear the virus, the innate immune responses will be strengthened, which cannot eliminate the virus effectively and even lead to systematic inflammation responses with the uncontrolled release of inflammatory cytokines. According to recent studies, severe and critically sick patients have a higher average age than mild cases (66 *vs*. 51 years), and more chronic cases are more likely to have multiple chronic disorders (72.3% *vs*. 37.4%). Due to the weakening of immune functioning, it takes longer for elderly patients and those with chronic diseases to establish efficient adaptive immune responses. These patients rely solely on their innate antiviral immune responses in the first stages of infection, which increases the risk of cytokine storms, severe disease onset, and mortality (Wang et al., 2020a). Although the kinetics of SARS-CoV-2 responses matches models of antiviral immune induction, it remains unclear if ongoing viral replication or immunological dysregulation causes immune hyperactivity. Pyroptosis is a massive inflammatory, caspase-1-dependent form of program cell death that happens regularly in response to internal cell pathogen infection (ICPI). It may play a role in the development of COVID-19. Rapid viral replication can increase pyroptosis, which can lead to a major release of pro-inflammatory mediators. Viral escapes to circumvent antiviral immunity and inherited or acquired weaknesses in host defense may restrict antiviral therapy, leading to abnormal immune activity and cytokine storms. As a result, the enhanced innate immune response could play a role in COVID-19/cytokine storm production (Figure 5) (Bergsbaken et al., 2009).

The secretion of numerous pro-inflammatory cytokines by neutrophil cells, monocyte cells, macrophages, and dendritic cells is referred to as a “cytokine storm.” Pulmonary immunopathology is a result of this process. Th1, Th17, and CD^+^ T cells may be stimulated, exacerbating lung damage. By lysis of tumor cells, the cytotoxic T lymphocytes (CTLs) contribute to antiviral therapy. Antibodies specific to viruses are produced by B cells, and they neutralize viruses (Hosseini et al., 2020).

#### 2.4.2 ACE-2 and its downstream pathway deregulation

The SARS coronavirus is engulfed by the angiotensin-converting enzyme 2 (ACE-2) receptor, which is required for cell fusion. ACE-2 is found in almost all organs and systems and plays a significant physiological role. ACE-2 also plays a protective role in various illnesses, including acute and chronic pro-inflammatory conditions. However, ACE-2 downregulation by the overactivation of the angiotensin-II/AT1R pathway and the harmful effects of angiotensin-II (Ang-II) caused by the SARS-COVID-19 spike protein could explain the multi-organ dysfunction in ill persons. In COVID-19, the role of Ang-II in the development of macrophage activation syndrome (MAS) and the cytokine storms are discussed in greater depth in the following paragraphs. In this analysis, we investigated the most current research developments in treatment strategies that primarily aim to decrease Ang-II-induced side effects instead of virus proliferation (Banu et al., 2020). As a vital counter-regulator, ACE-2 predominantly catalyzes the breakdown of Ang-II to maintain the homeostasis of the RAAS (Datta et al., 2020). ACE-2 has been already identified as a functional hosting receptor for SARS-CoV-2. Several parameters, including age, sex, ethnicity, medication, and several co-morbidities, were associated with changed ACE-2 expression and infection severity and development (e.g., heart attack disease, metabolic syndrome, and pulmonary cancer) (Liang et al., 2020).

**FIGURE 6:**
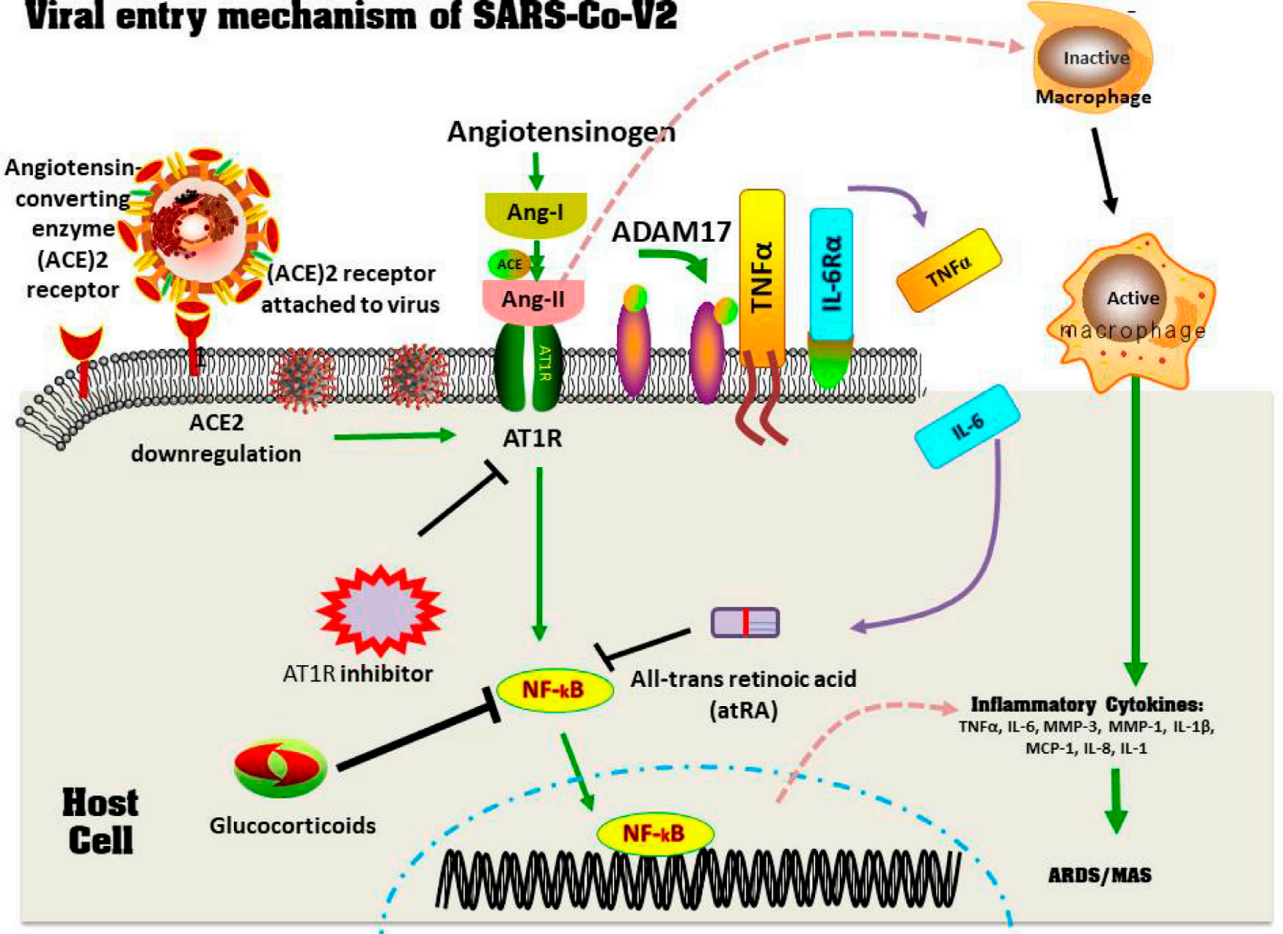
The emergence of MAS and ACE-2 Downregulation in a COVID-19 patients. Through the spike protein, the viruses accesses the host cell by connecting with the ACE-2 active site. Later, the virus suppresses ACE-2 impression, which causes Ang II to increase. Ang II is a RAS pathway product formed by the breakdown of Ang I by the enzyme ACE. Through NF-κB signaling, increased Ang-II binds with it's own receptor AT1R, modulating the gene expression of several inflammatory cytokine productions. This Ang-II/AT1R connection also affects macrophage responses, which results in the production of inflammatory cytokines productions, leading to ARDS or MAS. Furthermore, some metallo-proteases, such as ADAM17, convert these proinflammatory cytokines and ACE-2 receptors factors to a dissolved form, that aid inside the removal of surfaces ACE-2’s protective mechanism and that may contribute to SARS development. Therapy using glucocorticoids, AT1R blockers, as well as all-trans retinoic acid suppresses NF-κB regulation, which reduces cytokines storms and improves SARS-CoV2 disease severity (Based on Banu et al., 2020. Copyright. Elsevier 2020).

Recent studies have shown besides ACE-2 being a viral-binding receptor in COVID-19, it may play a role in modulating immunological responses (Catanzaro et al., 2020). In lung cancer, Chen and his coworkers discovered that the impression of many axes “mir-125b-5p-ACE-2-IL-6” in (mir-125b-5p) suppressed the impression of IL-6 by encouraging the upregulation of ACE-2 (Morassi et al., 2020). ACE-2 activity (mRNA levels and enzymatic activities) on the surfaces of host cells was significantly decreased after becoming joined by SARS-CoV-2, and IL-6, a downstream effector inside the number of fatalities receptor signaling cascade, could affect the immune response system. Cytokine storms and pneumonia may be increased as a result of the deregulation of ACE-2 induced by SARS-CoV-2, and blocking the upstream regulation mir-125b-5p could provide a novel strategy to manage COVID-19 (Figure 6) (Morassi et al., 2020).

More intriguingly, the SARS-CoV spike protein suppresses the ACE-2 impression, resulting in an overproduction of Ang-II (the downstream of ACE-2) by the connected enzyme ACE (Imai et al., 2005). Likewise, SARS-CoV-2 would downregulate ACE-2 receptors, resulting in an overproduction of Ang-II, which could be a plausible explanation for the cytokine storms in SARS/COVID-19 (Catanzaro et al., 2020). SARS-CoV-2 occupies ACE-2 molecules on the cell surface, and Ang-II rises as a result of reduced ACE-2-mediated degradation (Catanzaro et al., 2020). The accumulating Ang-II stimulates the IL-6 amplifier through greater activation of the nuclear factor kappa B (NF-κB) system and then the IL-6/STAT3 (transcript class 3 signaling transducers and stimulators) trajectories and SARS. CoV-2 super-activates NF-κB *via* diagram recognition receptors (DPRs) (Hirano and Murakami, 2020). This strong positive correlation is reinforced by IL-6, which results in out-of-control cytokine release. More importantly, IL-6 is a key marker of cellular aging, and the age-related strengthening of the IL-6 amplifier could be linked to COVID-19 mortality (Figure 6) (Murakami et al., 2019; Hirano and Murakami, 2020).

Through the spike protein, the virus accesses the host cell by connecting with the ACE-2 active site. Later, the virus suppresses the ACE-2 impression, which causes Ang-II to increase. Ang-II is a RAS pathway product formed by the breakdown of Ang-I by the enzyme ACE. Through NF-κB signaling, increased Ang-II binds with its own receptor AT1R, modulating the gene expression of several inflammatory cytokine producers. This Ang II/AT1R interaction also influences the macrophage activation that in turn produces the inflammatory cytokines Thereby, inducing ARDS or MAS. Furthermore, some metalloproteases, such as ADAM17, convert these pro-inflammatory cytokines and ACE-2 receptor factors into a dissolved form that helps remove ACE-2’s surface protective mechanism and that may contribute to SARS development. Therapy using glucocorticoids, AT1R blockers, and all-trans retinoic acid suppresses NF-κB regulation, which reduces cytokine storms and improves SARS-CoV-2 disease severity (Banu et al., 2020).

## 3 Inhibiting cytokine storm using approved drugs are key in COVID-19

**FIGURE 7:**
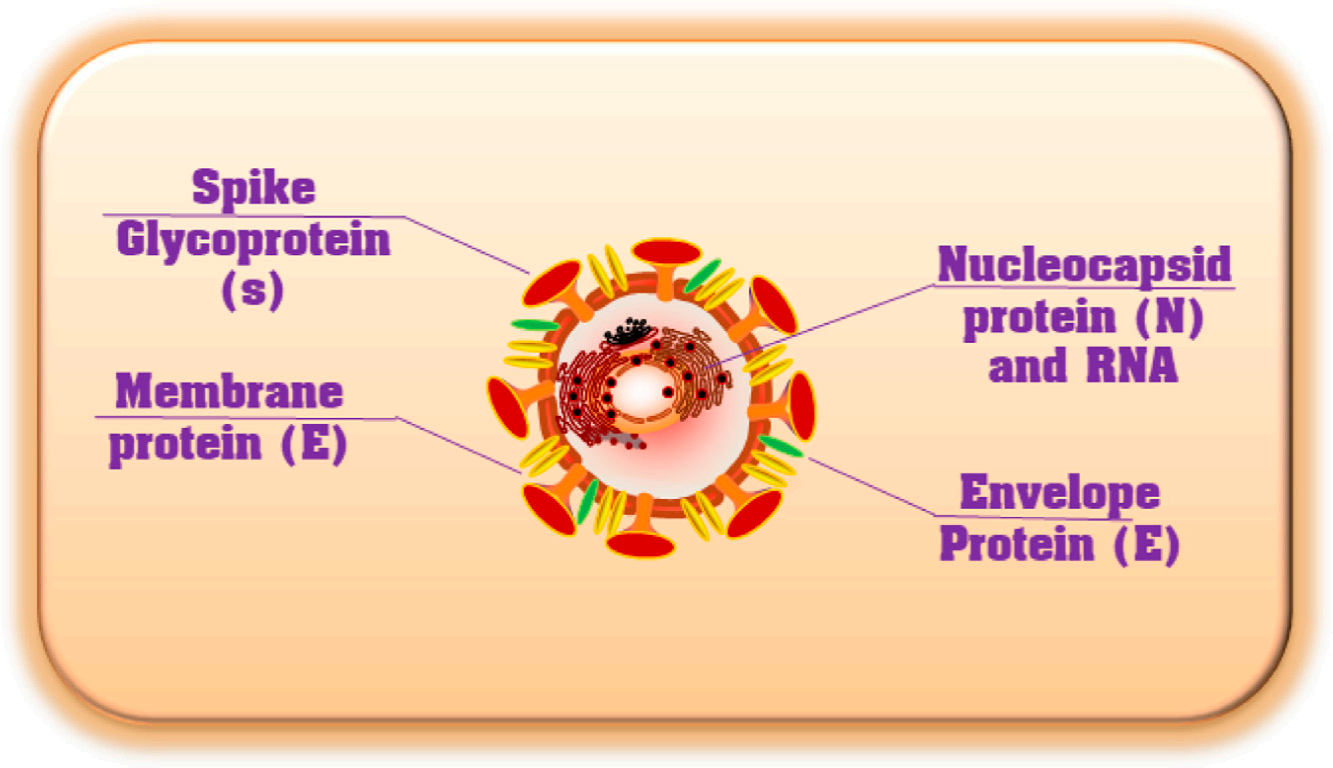
The Structure of COVID-19.

The SARS-CoV and MERS-CoV outbreaks should be used to gain significant information and insights into how to properly treat COVID-19 pneumonia patients. SARS-CoV-2 infection progresses through three stages: early infection, pulmonary phase, and hyper-inflammation phase (Siddiqi and Mehra, 2020). Targeted treatments are urgently needed to prevent cytokine storms, and the primary infection with minimal or moderate symptoms is the critical time for aggressive therapies to control subsequent degeneration (Table 1, Figure 7) (Siddiqi and Mehra, 2020).

**TABLE 1:** Structural of SARS/COVID-19.

Protein structural	Function of proteins
Spike protein (S)	Facilitates the interaction between COVID-19 and host cells ACE-2
Membrane protein (M)	Abundant protein that defines the shape of the viral envelope and plays a vital role in the formation of virus particles
Envelope protein (E)	Interacts with M proteins to form the envelope of the virus
Nucleoprotein (N)	Interacts with the viral RNA genome and assist in RNA synthesis and folding

Direct cell damage due to COVID-19 can be reduced with antiviral medications that block virus transmission and kill virus reproduction, whereas cytokine storms induced by the virus can be resisted with immunoregulatory therapies that inhibit hyper-activated inflammatory responses (Liu et al., 2020a). Several clinical trials started to look into possible treatments to manage a cytokine storm in COVID-19 cases, primarily focusing on direct cytokine inhibition and immunomodulatory medicines.

### 3.1 IL-6/IL-6R with different inhibitors

**FIGURE 8:**
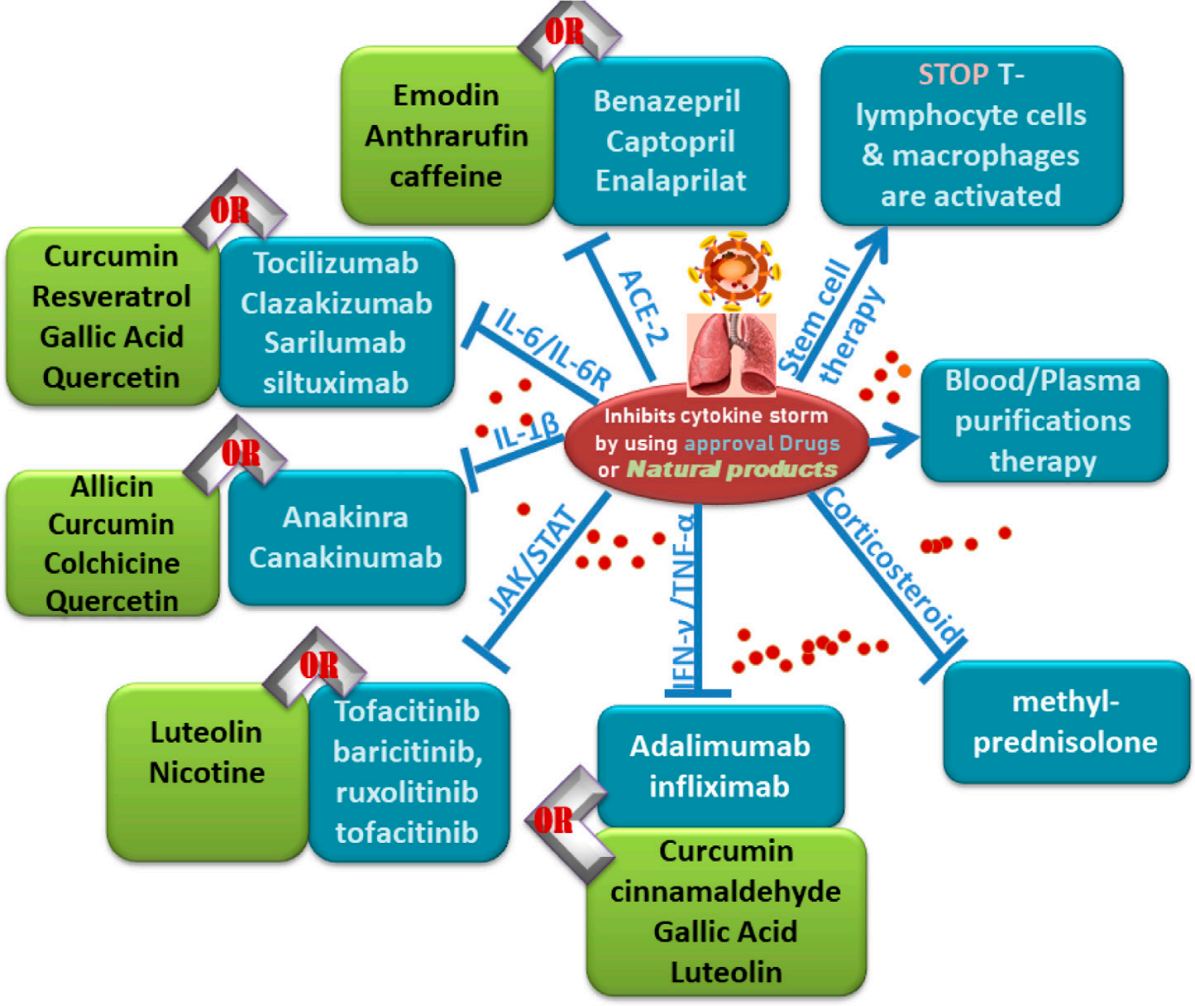
Follow charts: Different Inhibitors of cytokine storms by using approved drugs or natural products.

One meta-analysis of IL-6 concentration levels found that patients with complicated COVID-19 had 2.9-fold higher levels than those with uncomplicated disease (Coomes and Haghbayan, 2020). Another meta-analysis found a link between IL-6 levels and severity (Ulhaq and Soraya, 2020), indicating that the IL-6 level was a perfect predictor of poor prognosis in COVID-19 (Coomes and Haghbayan, 2020). In a number of ongoing COVID-19 clinical trials, IL-6/IL-6R inhibitors were employed preliminarily, and further inter-clinical trials are presently underway (Table 2; Figure 8). Tocilizumab (a type of IL-6 receptor inhibitor, IL-6R), first licensed to rheumatoid disorders, could successfully cure iatrogenic cytokine storms in patients with hematological malignancies produced by CAR-T therapy (Grupp et al., 2013).

**TABLE 2:** Side effects drug–immunosuppressant in protocol COVID-19.

Some drugs	Side effects drug–immunosuppressant
Lopinavir/ritonavir	GI upset, including N/V, skin rash, hypercholesterolemia, increased serum TGs, increased liver enzymes, diarrhea, abdominal pain, dysgeusia, and URT infection
Chloroquine/hydroxychloroquine	Retinopathy and maculopathy (occurred in long-term use), cardiomyopathy resulting in heart failure, QT interval prolongation, increased liver enzymes, bone marrow suppression (rare), hemolytic anemia, hypoglycemia, GI upset, occasional headaches, dizziness, and loss of appetite
Umifenovir	No data available
Ribavirin	Headache, fatigue, loss of appetite, diarrhea, abdominal pain, dyspepsia, neutropenia, anemia, lymphocytopenia, hemolytic anemia, increased serum bilirubin, musculoskeletal pain, influenza-like symptoms, and URT infections
Remdesivir	Rash, diarrhea, hypotension, and increased liver enzymes
Interferons	Fatigue, headache, chills, depression, malaise, neutropenia, granulocytopenia, leukopenia, anemia, thrombocytopenia, increased serum AST, ALT, and ALP, dyspnea, and cough
Tocilizumab	Hepatotoxicity, increased serum ALT and AST, injection-site and infusion-related reactions, increased risk of URT infections, neutropenia, leukopenia, and thrombocytopenia

Tocilizumab improved fever control and respiratory functioning in 21 severe COVID-19 patients in a Chinese clinical study (ChiCTR2000029765). All individuals, including two critically ill people, started to recover and were released from the hospital (Xu et al., 2020). Roumier et al. (2020) presented that tocilizumab lowers compressive respiratory requirements (OR = 0.43) and the risk of subsequent ICU admissions (OR = 0.18) in 30 COVID-19 cases. Toniati et al. (2020) presented a single-center trial in Italy with a hundred patients with COVID-19, revealing that tocilizumab treatment was effective, long-lasting, and linked to important clinical improvement. A thorough review and meta-analysis of observational data found decreased mortality in COVID-19 patients receiving tocilizumab (Toniati et al., 2020). Furthermore, despite a delayed antiviral therapy induced by a higher initial viral load, IL-6 destruction with tocilizumab did not affect viral-specific antibody levels, showing tocilizumab’s safety for COVID-19 patients (Masiá et al., 2020).

Sarilumab is another type of IL-6R inhibitor studied in SARS-CoV-2, which could indicate a potential therapeutic advantage of early intervention with IL-6-modulatory COVID-19 treatments (Montesarchio et al., 2020). Clazakizumab is a monoclonal antibody against human IL-6. Several studies found that severely ill SARS/COVID-19 patients who took tocilizumab or sarilumab showed better results and decreased mortality rates (Gordon et al., 2021; Gupta et al., 2021). Conversely, other research on the efficacy of tocilizumab or sarilumab has produced mixed findings, with the medications failing to lower the risk of intubation or mortality in COVID-19 patients in many clinical trials (Lescure et al., 2021; Rosas et al., 2021). On 24 June 2021, tocilizumab was approved as a treatment for COVID-19 in hospitals. This decision was based on the outcomes of big clinical trials on tocilizumab (Horby et al., 2021). The EUA is only licensed for treating certain hospitalized cases already treated with corticosteroids and requiring respiratory assistance. The medicine is not approved as a general COVID-19 treatment.

Both tocilizumab and sarilumab enhanced survival in COVID-19-positive critically ill patients in the ICU (Gordon et al., 2021; Gupta et al., 2021). Furthermore, in hospitalized COVID-19 cases who required oxygen and had evidence of inflammation, tocilizumab reduced the mortality rate within 4 weeks by an average deviation of 4% compared to standard care; this finding was based on COVID-19 cases that required oxygen and had indications of inflammatory response. Tocilizumab reduced the time patients stayed in hospital care by 50% to approximately 57% (p = 0.00010) (Horby et al., 2021). The best evidence that tocilizumab treatment aids COVID-19 cases who are hospitalized was found by Hwang et al. (2022). The WHO also recommends tocilizumab and sarilumab in combination with corticosteroids to treat severe COVID-19. Tocilizumab used for the treatment of COVID-19 reduced the risk of death within 28 days by an absolute difference of 4% compared to normal care in hospitalized patients; this finding was from COVID-19 patients who required oxygen and had evidence of inflammation. Tocilizumab significantly reduced the time patients stayed in hospitals, increasing the probability of discharge within 28 days from 50% to 57% (p = 0.0001) (Horby et al., 2021). Hwang et al. (2022) offered the most conclusive evidence that tocilizumab medication helps hospitalized COVID-19 patients. In addition, tocilizumab and sarilumab, in combination with corticosteroids, are recommended by the WHO to treat severe COVID-19. According to the study (NCT04322188), patients with rapidly developing COVID-19 respiratory pneumonia who require supplemental oxygen may benefit from siltuximab (IL-6R monoclonal antibody) therapy due to lower death and chemokines hyper-inflammation in Figure 5.

### 3.2 Blocking of the IL-1β family

In cytokine storms, three IL-1 family cytokines are important (IL-18, IL-1β, and IL-33), with the blocking of IL-1β to prevent cytokine storms from being of particular relevance (Vardhana and Wolchok, 2020). The FDA and the European Drug Administration (EDA) have approved anakinra, an IL-1β receptor antagonist inhibiting IL-1α and IL-1β activity, for the treatment of rheumatism, young rheumatoid arthritis with the systemic onset, and familial Middle Eastern fever (Ben-Zvi et al., 2017). Anakinra could also be administered to treat infection-induced cytokine storms, which improves the survival rate of patients with severe sepsis (Shakoory et al., 2016). Anakinra has a shorter half-life than other cytokine blockers, making it safer and more appropriate for severe and critically ill patients.

According to a case series trial (NC-T04324021), large doses of injectable anakinra diminish inflammatory responses and are linked to progressive improvement in pulmonary function in severe COVID-19 sufferers (Cavalli et al., 2020). Other clinical trials testing anakinra’s usage in COVID-19 are currently ongoing (e.g., NCT-04330638, NCT-04341584, and NCT-04339712). Canakinumab, a monoclonal antibody that targets IL-1β specifically, was investigated as a COVID-19 therapy. Canakinumab was found to be safe, well-accepted, and associated with a rapid decrease in systemic inflammation and improvement in cardiac and respiratory functions (Shakoory et al., 2016). However, more randomized controlled trials are needed to increase the efficacy of anakinra and canakinumab usage in COVID-19 treatment.

### 3.3 JAK inhibitor

The Janus kinase/signal transducers and activators of the transcription (JAK/STAT) signaling system, which is a typical downstream effector journey for many cytokines, can inhibit many specific cytokines at the same time when JAK/STAT was inhibited (Peterson et al., 2020). Potential adverse effects, such as an increased risk of pulmonary edema (PE), elevated liver enzymes, hematological disorders, and a loss in antiviral response, must be considered (Peterson et al., 2020). Tofacitinib, baricitinib, and ruxolitinib are among the JAK inhibitors currently investigated to determine whether they can treat COVID-19 (e.g., NCT-04332042, NCT-04348695, NCT-04321993, and NCT-04348695) (Peterson et al., 2020). Baricitinib and ruxolitinib are specific inhibitors of JAK1/JAK2, a protein implicated in various cellular signals, including the pro-inflammatory IL-6, that function as immunomodulators by lowering cytotoxic T lymphocytes while increasing regulatory T cells (Modi et al., 2019; Zhang et al., 2020c).

All clinical and pulmonary function parameters improved significantly in the baricitinib-treated group compared to the baseline in a phase 2 and 3 clinical study (NCT-04358614), with no viral diseases or cardiac or hematologic side effects (Vardhana and Wolchok, 2020). Although tofacitinib is a specific JAK1/JAK3 inhibitor, it can be begun in patients who are not already using it and be continued in those using it during a pandemic (Jacobs et al., 2020).

### 3.4 IFN-γ inhibitor/TNF-α inhibitor

TNF-α is a cytokine that regulates IL-6 expression and is involved in various inflammatory disorders. In contrast to anti-IL-6 therapy, anti-TNF therapy was demonstrated to suppress the production of various inflammatory cytokines, including IL-6, IL-1β, and GM-CSF (Charles et al., 1999). Additionally, prior studies have found higher levels of TNF in the blood and tissues of COVID-19 patients (Del Valle et al., 2020). Anti-TNF-α medication has lately been proposed as a strategy for lowering inflammation in COVID-19 patients after IL-6 blocking showed little efficacy (Steeland et al., 2018). Anti-TNF-α drugs, such as adalimumab or infliximab, can reduce the death rate in COVID-19 sufferers, according to preliminary clinical data (Stallmach et al., 2020). Until recently, limited clinical trials have been conducted on infliximab (NCT-04344249, NCT-04425538, NCT-04593940, and NCT-04734678) and adalimumab (NCT-04705844) in COVID-19. In COVID-19, no clinical evidence or registered clinical trials have evaluated the possibility of IL-18 and IL-33 blockers. TNF-a and IFN-γ are also important inflammatory cytokines that provide good targets for cytokine storm control [69], and clinical trials to evaluate these blockers in COVID-19 (ChiCTR-2000030089 and NCT-04324021) are continuing (Blaszczak et al., 2020; Cavalli et al., 2020).

### 3.5 Corticosteroid hormonal therapy

Corticosteroids, a kind of steroidal hormone, inhibit histone acetyltransferase (HAT) activity and recruit histone deacetylases-2 (HDAC-2) activity to downregulate inflammatory genes by interacting with the cytoplasmic corticosteroid receptor (Darwish et al., 2011). Corticosteroids are routinely used to control cytokine storms as a result. According to data from a short trial (Yang et al., 2020b), early administration of low or medium doses of methylprednisolone had a beneficial effect on sick people with severe COVID-19. In Iran, a single-blind, randomized, controlled trial (IRCT-20200404046947N1) found that the methyl-prednisolone pulse could be an effective treatment for COVID-19 patients in the pulmonary phase who were hospitalized (Edalatifard et al., 2020). For the treatment of severe cases, clinicians must master the time and dose of corticosteroids, mainly before infection occurs. Patients who already have hypoxemia for various reasons or who take glucocorticoids on a daily basis for other chronic diseases should be treated with the utmost caution (Ji et al., 2020; Nelson et al., 2021).

### 3.6 Blood/plasma purification therapy

Blood/serum filtration systems, such as serum exchange, adsorption, perfusion, and blood/plasma purification, eliminate inflammatory components and thereby minimize tissue damage caused by hyper-activated inflammation mediators (Ji et al., 2020). Li’s team presented a technique that artificially purifies the blood in the liver and could quickly eliminate inflammatory responses; stop cytokine storm; favor fluid, electrolyte, and acid/base homeostasis; and improve therapeutic efficacy in critical patients (Zhang et al., 2021). In a limited experience, Ma et al. (2020) found that three cases of COVID-19 that had serum/plasma purification therapy were effective and tolerable. One case report (Wang and Hu, 2020) described the successful recovery of a severely ill patient. Based on previous experiences with SARS and MERS, Yang and his team developed a blood (serum/plasma) purification protocol for patients with COVID-19 sufferers that included four main steps: 1) deciding whether chronic COVID-19 patients need blood purification; 2) prescribing a plasma purification treatment for COVID-19 patients; 3) evaluating and adjusting blood purification measures; and 4) determining when blood purification should be stopped (Liu et al., 2020b; Yang et al., 2020c). Finally, to ensure a safe and efficient treatment, probable side effects of blood-purifying therapy (e.g., thrombocytosis, allergies, hemorrhage, and air edema) must be identified and handled as soon as feasible (Liu et al., 2020b).

### 3.7 Stem cell therapy

Mesenchymal MSCs also prevent T lymphocyte cells and macrophages from becoming abnormally activated and encourage their development into regulatory T cells and macrophages anti-inflammatory (Yagi et al., 2010). MSCs reduce the development of cytokine storms by inhibiting the release of pro-inflammatory cytokines (Yagi et al., 2010). Many clinical trials involving intravenous MSC delivery in COVID-19 patients were officially filed (www.clinicaltrials.gov). However, most of them are still recruiting participants. Zhao’s team recently published the outcomes of seven chronic and critically sick COVID-19 patients who had MSC transplantation therapy, demonstrating improved prognosis and successful cytokine storm avoidance with no apparent side effects (Leng et al., 2020). Other stem cell therapies, such as stem cells generated from human menstrual blood (ChiCTR-2000029606) and embryo stem cells, are also in clinical testing (ChiCTR-2000031139). Not only can stem cells (MSCs) self-renew and differentiate in multiple directions, but also they have anti-inflammatory and immune-regulatory properties (Orleans et al., 2020).

## 4 Natural immunosuppressants as adjuvants in managing COVID-19 to target the cytokine storm

With no specific antiviral drugs to cure the virus and no vaccination to stop it, the COVID-19 outbreak generated a worldwide health crisis with massive side effects. Although some people infected with SARS-CoV-2 had a well-coordinated immune response and recovered, others had a defective immune response that led to significant consequences such as ARDS, sepsis, and MOF, all linked to increased death and disability.

According to previous studies, a huge cytokine/chemokine syndrome release dubbed “cytokine storms” was discovered in patients with a malfunctioning immune response. As a result, these patients had greater levels of pro-inflammatory/modulatory cytokines (e.g., IL-β1, IL-2, IL-4, IL-6, IL-7, IL-9, IL-10, IL-12, IL-13, IL-17, TNF-α, INF-ℽ, G-CSF, GM-CSF, M-CSF, and HGF) and chemokines (CXCL-8, MCP-1, IP10, MIP-1, and MIP).

**FIGURE 9:**
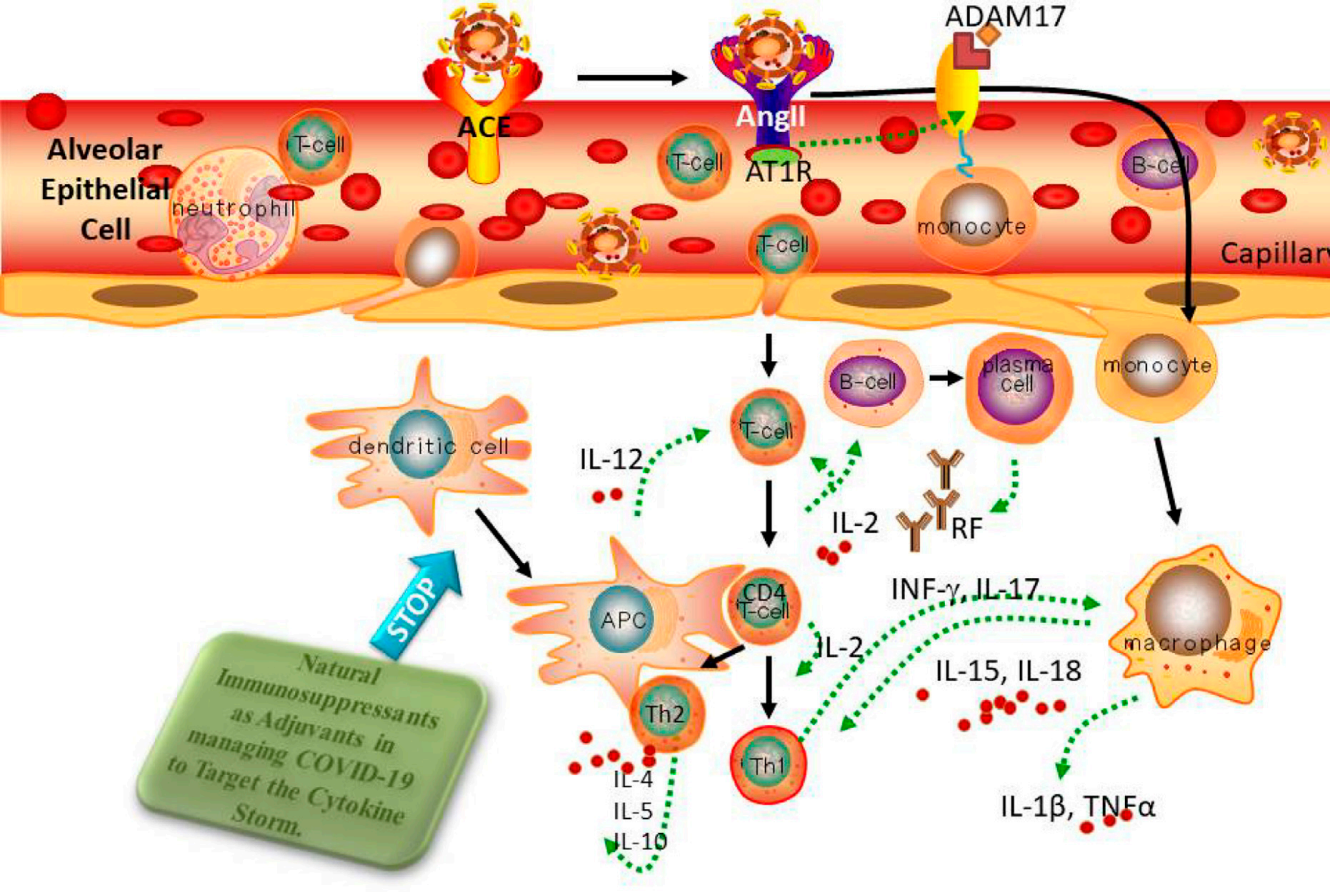
Blocking the cytokine storm is a unique, potential natural treatment technique for reducing the excessive influx of cytokines seen at the infected site.

Immunomodulatory activities have long existed in natural products, crude extracts, and metabolites. Additionally, the ability of several plant-derived bioactive constituents to reduce the cytokine storms associated with inflammatory disease has been investigated. The CSS seen in COVID-19 sufferers is briefly discussed in this review, with an emphasis on several natural-immunosuppressant drugs obtained from plant extracts that can help reduce the cytokine storms seen in severe COVID-19 sufferers (Figure 9; Table 3).

**Table 3:** Chemical structure of Natural compound list as Immunosuppressants as Adjuvants in managing COVID-19 to Target the Cytokine Storm. The remainder of this table can be found in the Supplementary Materisl.

Natural metabolites, Groups	Sources and part was used	Therapeutic properties/	Mode of Action/ Docking studies	Clinical Trials
**1. Curcumin** [(1E,6E)-1,7-bis(4-hydroxy-3-methoxyphenyl) hepta-1,6-diene-3,5-dione] , Polyphenol group. 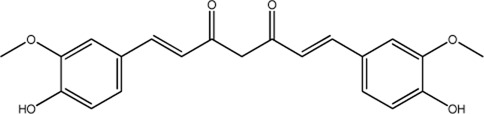	*Curcuma longa*, Zingiberaceae family the rhizomes part are used	Natural antioxidant , Anti-inflammatory, Neuroprotective, Hepatoprotective , Stop tumour cells, and Immunomodulator (Hewlings and Kalman, 2017, Hamed et al., 2019).	- It used a decrease secretion of proinflammatory cytokines/chemokines such as (IL-1β, IL-6, IL-2, IL-12, TNFα, IFNγ, IP10, CXCL8, MCP1, and MIP1α) in monocyte culture exposed to preeclamptic serum (Rahardjo et al., 2014). - It blocked chemokines NF/κB pathway by IκBα hydrolysis or stimulating AMPK to damage the NF/κB pathway by focusing on p65 and p50. (Sordillo and Helson, 2015, Shimizu et al., 2019, Liu and Ying, 2020). - It performs as an immunosuppressant by decreasing IKKβ activation.(Sordillo and Helson, 2015, Shimizu et al., 2019, Liu and Ying, 2020). Curcumin was suppressed the production of the IL-18/ in LPS-expression murine macrophage such as RAW264.7 cells(Yadav et al., 2015).	- In COVID-19 patients 40 sick clinical from Tabriz University of Medical Sciences' Imam Reza Hospital was conducted in Iran. The nano-curcumin160 mg in capsules daily for fourteen days reduce the levels of hypperinflammatory cytokines: (IL-6 & IL-1β) other hand, had no effect on IL-18 mRNA expression or TNFα levels (Babaei et al., 2020, Valizadeh et al., 2020, Zahedipour et al., 2020). - The clinical study into curcumin's potential as an immunomodulatory adjuvant to lessen the cytokines storms, hyperinflammation, and ARDS associated with SARSCoV-2 infection. (Sordillo and Helson, 2015, Liu and Ying, 2020).
**2. Resveratrol** 5-[(E)-2-(4-hydroxyphenyl) ethenyl]benzene-1,3-diol], phytoalexin polyphenol group. 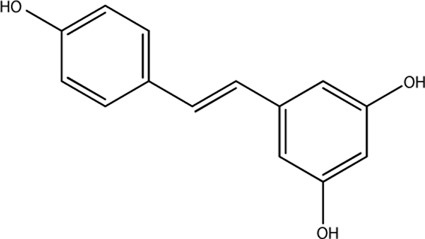	Grapes, cranberries, blueberries, mulberries, peanuts, jackfruit, soy, wines.	Natural immunomodulator (Malaguarnera, 2019), antioxidant, antimicrobial(Attia et al., 2021), chemotherapeutic, anti - inflammatory properties, antiviral, anti-aging, and life-prolonging properties. (Bansal et al., 2018).	- It inhibited the secretion of production. (IL-2, MCP1, IL-8, and IFNγ) In EV71-infected RD cells(Zhang et al., 2015) and HTLV-1 cells (Rieder et al., 2012) by splenic lymphocytes and inhibited the production of TNFα, IL-12 and IL-17 by peritoneal macrophages in lung inflammatory cells with a reduction in NF-κB and IκBα expression(Gao et al., 2001). - Resveratrol's **treated in pig's** had ability to reduce IKK activity coincides with its inhibitory effect on the NF-κB pathway therefore influencing humoral immune responses(Fu et al., 2018). - **In a rabbit** model of acute pharyngitis, resveratrol therapy reduced protein expression of IL-1β , IL-18,TNFα, IL-6, MIP2, and p-NF-κB and elevated while suppressing TLR4 and myeloid differentiation primary response protein 88 expression (Zhou et al., 2018). - Phase 2 /Resveratrol’s multimodal antiviral, anti-inflammatory, and antioxidant properties as well as its ability to upregulate ACE_2_ receptors could be helpful in reducing the clinical effects of COVID.	-Resveratrol inhibited the release of inflammatory cytokines and chemokines (GM-CSF and CXCL8) by activated alveolar macrophages from COPD patients, table (4) (Culpitt et al., 2003). -Resveratrol used as an additional treatment for SARS-CoV-2 infection in order to minimise inflammation by reducing the cytokine storm (Marinella, 2020). Clinical.Trials.gov: NCT04400890, NCT04799743, NCT04542993
**3. Gallic Acid and polygallic acid** 3,4,5-trihydroxybenzonic acid, phenolic metabolite. 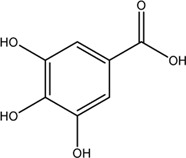	Many fruits as blackberry, blueberry, strawberry, grapes, cashew nut, plumsmango, walnut, hazelnut, and tea.	Natural antioxidants, antimicrobial, antifungal, anticancer, anti-ulcerative colitis and antiinflammatory properties (Zhu et al., 2019).	- Inhibits in TNBS-stimulant, the hyper-inflammatory cytokine ( IL-1/TGF/IL-6/IL-17/IL-12/IL-23/TNFα), chemokines (CCL2 & CCL7) and NF-B pathway (Zhu et al., 2019). - Inhibits the Th2 cells cytokine as (IL-4 & IL-5) but not the Th1 cells cytokines IFNγ in anti-CD3-stimulated/spleen cells, according to (Kato et al., 2001). - Block PMA adds A23187-induced IκBα destruction and p65 in NF-κB nuclear dislocation. - In stimulated HMC-1 cells, gallic acid and polygallic acid administration dramatically reduced the release of both of these cytokines (Kim et al., 2006) - Gallic acid could be utilized as an immunosuppression adjuvant to decrease inflammatory cells selectively, so target the cytokine release syndrome seen in SARS-CoV-2 patients without impairing the owner's ability to produce interferon’s.	-In high glucose-induced human monocytes (THP-1 cells), GA treatment reduced NF-κB signaling and reduced proinflammatory cytokines as IL-6 production (Lee et al., 2015) In human mast cells (HMC-1) was decrease the production of pro-inflammatory cytokine (IL-6 & TNFα). PMA with A23187 were utilized to activate HMC-1 cells, leading to the release of TNFα and IL-6.
**4. Luteolin** [2-(3,4-dihydroxyphenyl)-5,7-dihydroxychromen-4-one] a naturally occurring flavonoid 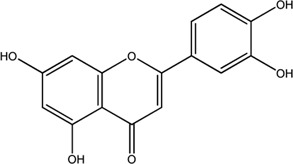	Many vegetables (celery, broccoli, parsley, peppers, carrots, and cabbages) as well as in fruits (apple skins), in flowers (chrysanthemum), and others medicinal herbs contain this compound.	It's a strong antioxidant that stops tumour cells from proliferating and reduces metastasis. It also functions as an anti-proinflammatory and immune function modulator. (Lin et al., 2008).	- Inhibition production of TNFα, IL-1β, IL-6, NF-B, STAT1, in rats with MSU-induced inflammation(Lodhi et al., 2020) and IRF1 suppression (Kao et al., 2011). - luteolin administration on PMA plus A23187-induced HMC-1 cells resulted in considerable reduction of (TNFα, IL-6, CXCL8 & GM-CS), and NF-κB activation suppression (Kang et al., 2010). - A reduce in AP1 transcription factor specific binding activity and block JNK phosphorylation, which had no effect on the LPS-induced increase in NF-κB DNA binding activity or even the LPS-induced degradation of IκBα. - luteolin pretreatment resulted in a dose-dependent reduction in mRNA level expression and the degrade release of IL-6, IL-8, &VEGF in human HaCaT and primary keratinocytes.	-Luteolin has wide antiviral action, block mast cells, and may block COVID/SARS-CoV-2 major protease (3CLpro) (Theoharides, 2020). - luteolin is a immunomodulatory properties essential natural medication to examine as a therapeutic treatment that could be further investigated as an adjuvant to reduce the cytokines storms seen in COVID-19 and potentially decrease SARSCoV-2 infection. More clinical trials in this area are needed to determine the safety, effectiveness and dose for COVID-19 therapy. - Luteolin suppressed NF-κB activity in human monocytes in hyperglycemic conditions, resulting in a considerable reduction in the release of IL-6 and TNFα. Luteolin significantly inhibited the generation of IL-1β, IL-6, TNFα, and IFNγ in human whole blood treated with LPS(Ribeiro et al., 2015). - Clinical.Trials.gov: NCT05311852
**5. Quercetin** Flavonoid as [2-(3,4-dihydroxyphenyl)3,5,7-trihydroxychromen-4-one]. 1-Prophylaxis: 500 mg/daily. Treatment: 1000 mg/daily 2-600 /daily for 1st week; 400 mg/daily for 2nd week Phase 3 3- Two tablet twice a day Phase 1, 4-400 mg/daily Phase 3 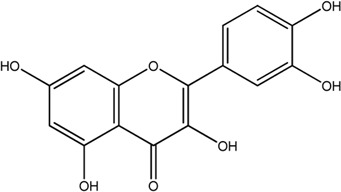	many plants including broccoli, red onions, eggplant, potatoes and green leafy vegetables including celery, lettuce; fruits including apples, citrus fruits, red grapes, tomatoes; berries include cranberries and raspberries.	Quercetin an excellent candidate for dealing with situations in which oxidative stress, inflammation, Rheumatoid arthritis, hypercholesterolemia, cardiovascular disease, anti-cancer (Li et al., 2016) and the immune system are involved.	- Reduce the secretion of hyper-inflammatory cytokine such as (TNFα, IL-6, G-CSF, GM-CSF, and VEGF), as well as chemokine (IP-10 and MCP-1), and increase the level of anti-inflammatory cytokine IL- 27 (Mehrbod et al., 2018). - In cultured blood peripheral mononuclear cells, it was also discovered to induce T-helper cells to give IFNγ and to down regulate Th2-cells derived IL-4 (Colunga Biancatelli et al., 2020). - Quercetin therapy considerably inhibited carrageenin-induced IL-1β production. - Inhibit the secretion of inflammatory cytokine is due to its inhibition of the activity of ERK & p38 MAP kinase, as well as the NF-κB/IκB signalling pathways (Tang et al., 2019). - Quercetin inhibited the activation of phosphorylated ERK kinase and p38 MAP kinase in LPS-stimulated RAW 264.7 cells. There was no discernible effect on JNK MAP kinase(Tang et al., 2019).	- Treatment with quercetin reduced the levels of IL-1β, TNFα, and IL-10 in patients with coronary artery disease, according to another study. This was related to transcriptional factor NF-κB activity being reduced (Chekalina et al., 2018). - Quercetin treatment (Oral supplementary) is comparatively safe, having no severe side effects; the only side effects detected in two out of thirty patients in a study were headache and brief peripheral paresthesia. - The use of quercetin as an additive to improve the immune response by promoting IFNs production and changing levels of pro-inflammatory cytokines could be a useful addition to current COVID-19 therapies. (Colunga Biancatelli et al., 2020). - All Clinical trial studied/ 15 number tell now: Clinical.Trials.gov: NCT04377789, NCT04861298, NCT04853199, NCT04578158
**6. Cinnamaldehyde and Cinnamic acid.** [(E)-3-phenylprop-2-enal] 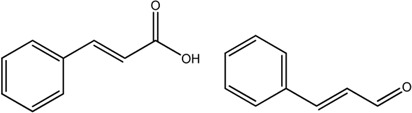	*Cinnamomum verum*, *Cinnamomum burmanii Cinnamomum zeylanicum, Cinnamomum cassia*, *Cinnamomum loureirii, Cinnamomum tamala*, &*Cinnamomum cordatum.*	anti-gastric ulcer, antioxidants, anti-inflammatory, anti-microbial, anti-yeast(Dorri et al., 2018), anti-platelet, and hepatoprotective properties that could help manage COVID-19 infection (Gautam et al., 2020). Cinnamon can thus be used as an antidote to both natural and chemical toxins.	- Cinnamon inhibits the release of IL-1β, IL-6, TNF-α, and nitric oxide (NO) molecules (Ho et al., 2013). - Reduced LPS-induced IL-8 secretion in THP-1 monocytes through altering the TLR-2 and TLR-4 signalling pathways(Schink et al., 2018).	- They have the ability to bind HSPA5 substrate binding domain *β* (SBD- *β*) with a binding energy of -6.25 *±* 1.10, potentially interfering with COVID/SARS-CoV-2 recognition and binding (Elfiky, 2021).
**7. Pavetannin C1 and Tenufolin** **Bioflavonoids groups** 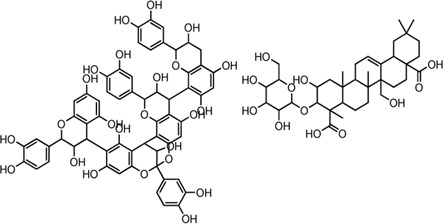	*Cinnamomum zeylanicum bark*	**:** antioxidants, anti-inflammatory (Dorri et al., 2018).	-Tenufolin and Pavetannin C1, have good affinity with the main proteases a binding energy as ( -8.8 and -7.3) and a high affinity with spike proteins of SARS-CoV-2 a binding energy as (-8.7 and -11.1), respectively (Prasanth et al., 2020, Sasidharan et al., 2022). .	Clinical trials have demonstrated its efficacy (Sasidharan et al., 2022).
**8. Allicin** [3-prop-2-enylsulfinylsulfanylprop-1-ene], called thiosulphate Group. 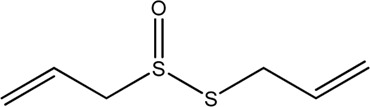	Garlic oil is the component that gives garlic its unique smell and flavour (*Allium sativum*). Allicin can also be found in the field onion (*Allium cepa*) and another Alliaceae species. (Borlinghaus et al., 2014).	antioxidant, antimicrobial, antiviral, antiinflammatory, antitumor and antidiabetic properties (El-Saber Batiha et al., 2020)	- Allicin inhibited TNF-α induced production of (IL-1β, CXCL-8, IP-10, and MIG in HT-29), NF-κB pathway suppresses the breakdown of IκB, resulting in a decrease in cytokine production (Lang et al., 2004). - Allicin therapy increased defense and survival in BALB/c mice after Plasmodium yoelii infection due to an increase in IFNγ production and proliferation of CD4^+^T-cells. - Allicin supplementation combined with tamoxifen treatment resulted in a significant drop in TNF-α levels in Ehrlich ascites carcinoma (EAC) cells, demonstrating its therapeutic effects as an adjuvant (Arreola et al., 2015).	- There were no side effects recorded in clinical trials examining the safety of allicin therapy and pharmacokinetics (with antiviral drugs in use) in COVID-19(Sharifi-Rad et al., 2019). - In a study with cultivated human umbilical vein endothelial cells, allicin reduced TNFα, CXCL8, NFB activity levels and LPS-induced inflammatory reactions (HUVECs) (Zhang et al., 2017). - Inhibits NF-κB signaling for decreased cytokines secretion, improve protection by raising IFNγ production for stronger improved antiviral defense, and promote CD4^+^ T-cell expansion therefore targeting lymphocytopenia, allicin is a highly promising immunomodulatory adjuvant which can be utilized in concert with antiviral medication in COVID-19 patients.
**9. Eugenol compound** 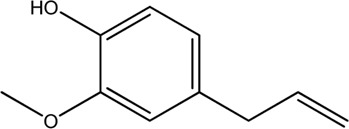 2-methoxy-4-prop-2-enylphenol	Clove buds (Syzygium aromaticum), tulsi leaves, cinnamon bark and leaves, pepper, turmeric, ginger, and natural herbs such as oregano, mace, basil, thyme, bay, marjoram, and nutmeg (Khalil and Rahman, 2017).	Anticancer, antioxidant, analgesic, antimicrobial, anticonvulsant, and antimicrobial properties, anti-inflammatory and antiviral activities, as well as being an immunomodulatory drug (Pramod et al., 2010, Dibazar et al., 2015).	- Eugenol inhibited the production of IL-6 and IL-10, IL-4 and IL-5, as well as NF-κB pathways, and thereby OVA-induced eosinophilia was guarded from lung cells study (Bachiega et al., 2012). - Eugenol also inhibited TNFα and IL-1β production as well as the signaling NF-κB pathways (ERK1/2 & p38- MAPK /in LPS-stimulated macrophages) (Barboza et al., 2018). - Eugenol enervated the inflammatory response in pig intestinal epithelial cells by dramatically lowering both CXCL8 and TNFα mRNA levels in an LPS-induced inflammatory model (Hui et al., 2020).	**-** It may be a suitable natural immunosuppressant that can be utilized in combination with antiviral medicines to suppress COVID-19's hyper-cytokinemia and hyper-inflammation.
**10. 6-shogaol and 6-gingerol compounds** [**6-gingerol,** (5S)-5-hydroxy-1-(4-hydroxy-3-methoxyphenyl)decan-3-one], [**6-shogaol,** (*E*)-1-(4-hydroxy-3-methoxyphenyl)dec-4-en-3-one] 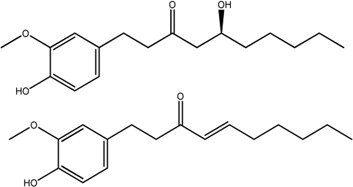	*Zingiber officinale* rhizomes belongs to Zingiberaceae family and traditional medicine from ancient times.	antioxidant, anti-metastatic, anti-inflammatory, anti-angiogenic, analgesic, anti-diabetic, and antipyretic properties. Furthermore, gingerol is reported to have anti-allergic, neuroprotective, immunomodulatory, and anti-carcinogenic activities. (Sharifi-Rad et al., 2017).	- In LPS-stimulated macrophages, 6-gingerol was decrease e production of cytokines IL-1β, IL-12, & TNFα and chemokine CXCL8 & COX2 without interfering with their antigen presentation function and AMPK activation(Tripathi et al., 2007, Sheng et al., 2020). - Inhibits NF-κB pathway being down-regulated as a result of an increase in phosphorylated IKBα and p65 downregulation (Saha et al., 2016).	- An *in silico* analysis found that 6-shogaol and 6-gingerol compounds have a high affinity of S-spike protein in SARS-CoV2 and ACE-2 receptor, suggesting that they could help reduce viral load and SARS-CoV-2 shedding in the nasal passage (Haridas et al., 2021). - After being used in traditional antiviral medication, 6-gingerol is a potent natural immunotherapeutic drug that could help control the cytokine storm seen in COVID-19.
**11. Melatonin** N-acetyl-5- methoxytryptamine, acetamides groups. 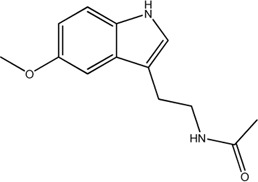	*Curcuma aeruginosa*, *Piper nigrum*, *Oryza sativa*, *Zea mays*, *Brassica hirta*, *Apium graveolens*, *Helianthus annuus*, *Foeniculum vulgare*, *Prunus amygdalus*, *Coriandrum sativum*, *Pimpinella anisum*, and Coffea arabica L. It has been found in several plant species from the Brassicaceae, Poaceae, Vitaceae, Rosaceae, and Apiaceae, families (Nawaz et al., 2016).	Antioxidant, anti-inflammatory, anti-viral activity, and anti-pro-inflammatory cytokines (Salehi et al., 2019).	- Melatonin inhibited NF-κB/STAT1/3STAT/GAS/, a major transcription factor as inflammatory cytokines (Min et al., 2012). - Melatonin decreases in serum levels of IL-6, TNFα, and IL-1β (Zhang et al., 2020b). - In a study of male C57BL/6 mice with cadmium-induced liver injury, melatonin treatment was discovered decrease response inflammatory cytokine secretion and block NLRP3 cells inflammasome activation (Cao et al., 2017). - Melatonin therapy effectively decreased the LPS- effected generation of many chemokine MCP1, CCL9, and CCL5 mRNA expression in BV2 murine microglial cells in another investigation. -	-Melatonin strongly inhibited IL-8 production in acrolein-stimulated human lung fibroblasts. (Kim et al., 2012). - According to a recent mechanistic investigation, melatonin treatment can effectively reduce the cytokines storms in COVID-19 by reversing aerobic glycolysis in system immune cells (Reiter et al., 2020). These findings support the use of melatonin as an immunosuppressive supplement to reduce the cytokines storms syndrome seen in COVID-19. (Zhang et al., 2020b). - All Clinical trial studied/ 11 number tell now: Clinical.Trials.gov: NCT04474483, NCT04409522, NCT04568863, NCT04530539, NCT04353128.
**12. Morphine and Codeine compounds** An opiate alkaloid groups **[morphine**, (4R,4aR,7S,7aR,12bS)-3-methyl-2,4,4a,7,7a,13-hexahydro-1H-4,12 methanobenzofuro[3,2-e]isoquinoline-7,9-diol)**]** binds to a specific the μ,δ,κ opiate receptors involved in modulating various activity of the brain and **[Codeine**,(4R,4aR,7S,7aR,12bS)-9-methoxy-3-methyl-2,4,4a,7,7a,13-hexahydro-1H-4,12-methanobenzofuro[3,2-e]isoquinolin-7ol)]. 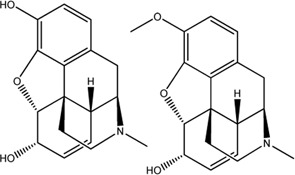	*Papaver somniferum*	Anti-diarrheal, analgesic, and antitussive effects. Morphine and codeine are thus potential immunosuppressant adjuvants for reducing the cytokine storm seen in COVID-19	- Morphine has decrease secretion of IFNγ, IL-6, IL-12, IL-1β, MCP 1, and TNFα in bronchoalveolar lavage fluids and pulmonary tissue of CB6F1 mice (Fukada et al., 2012) - In lung resident cells, morphine therapy suppressed the transcription factor NF-κB. A-stimulated splenocytes extracted from male Swiss mice, codeine treatment significantly reduced the generation of IL-2. - Docking tests demonstrated that morphine and codeine bind to ACE2 with a high affinity. This could theoretically limit cytokine release mediated by receptors. (Madera-Salcedo et al., 2011).	- Morphine's immunosuppressive action in relation to cytokines have been proven in clinical practice for over a century (Dinda et al., 2005). - **Ho**wever, more research is needed to investigate whether morphine or codeine should be used to treat COVID-19, because the time of morphine treatment affects cytokines secretion; Codeine has also been discovered to promote the synthesis of cytokines and chemokines by mast cells in vitro, and late treatment improves cytokine output. (Fukada et al., 2016). - All Clinical trial studied/ 2 number tell now: Clinical.Trials.gov: NCT04522037, NCT05381883.
**13. Nicotine** 3-(1-methylpyrrolidin-2-yl) pyridine, Alkaloid groups 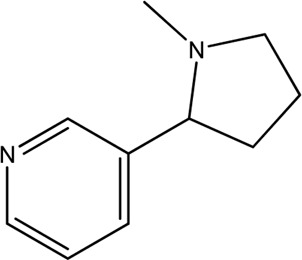	*Nicotiana tabacum,* Crassulaceae, Asclepiadaceae, and Solanaceae families.	It's a neuropsychiatric drug, an immunomodulator, as well as a drug for the peripheral nervous system. (Peter et al., 2021).	- The main mechanism of action involves the stimulation of a7-nAChRs onto inflammatory cells such as macrophages and neutrophils, which results in the suppression of NF-κB activation and, as a result, the release of pro-inflammatory cytokines and chemokines by these cells. - In a study with PBMC induced by HT-29 colon cancer cells, nicotine inhibited the production of (TNFα, IL-1β, IFNγ, and IL-2) but had no effect on the activity of IL-6. Nicotine therapy in PBMC driven by RKO colon cancer cells showed no significant effect on cytokine production (Djaldetti and Bessler, 2017). - Nicotine has been shown in studies to decrease the secretion of IL-1, IL-6, IL-12, INF, MIP1, and TNF. Nicotine decreases the phosphorylation of I-B, which reduces the transcriptional activity of NF-B (Piao et al., 2009)..	- It is used to treat ulcerative colitis in the hospital to reduce inflammation. Nicotine inhibits inflammatory cytokines by interacting through the cholinergic anti-inflammatory system through the a7-nicotinic acetylcholine receptor, as it is a cholinergic inhibitor (a7-nAChRs). IL-1β, and IL-6, TNF production are all inhibited by nicotine (Gonzalez-Rubio et al., 2020) - In human macrophages and splenocytes, nicotine reduces NF-κB and TNFα release caused by the LPS system. This inhibitory activity is related to nicotine's ability to stimulate JAK2 and STAT3 in macrophages, which is controlled through tristetrapolin (TTP) expression [172]. - Nicotine is also a widely available and well-recognized therapy. As a result, it is suggested that nicotine is a good adjuvant that can reduce the cytokine storm in COVID-19 and, in the short term, prevent growing mortality (Piao et al., 2009) - Clinical.Trials.gov: NCT04598594
**14. Piperine** (2*E*,4*E*)-5-(1,3-benzodioxol-5-yl)-1-piperidin-1-ylpenta-2,4-dien-1-one, alkaloid group 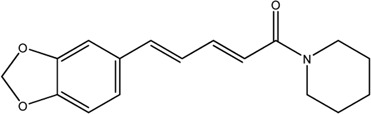	*Piper nigrum* L., Piperaceae species	Antibacterial, antioxidant, anti-metastatic, anti-inflammatory, and hepatoprotective properties, immunomodulator, and it renowned for increasing the bioavailability of medicinal drugs (Gorgani et al., 2017).	- In an ovalbumin-induced asthma model, piperine has been found to block the secretion of Th2 cells cytokine as (IL-4 & IL-5) (Kim and Lee, 2009). - Piperine blocks LPS-stimulated expression and secretion of hyperproinflammatory cytokine such as (IL-10, IL-6, IL-1β, & TNFα through inhibit NF-κB stimulation, and MAPK pathways (Dzoyem et al., 2017) ( Liang et al., 2016 ) (Zhai et al., 2016).. - **Docking studies:** As a result, piperine could be a promising therapeutic treatment not only for preventing virus adhesion to host cells, but also for preventing cytokines storms throw inhibiting the activity of the MAPK and NF-κappaB pathways, which leads to the release of pro-inflammatory cytokines (Maurya et al., 2020).	- Piperine therapy resulted in a significant decrease in the concentrations of (TNFα, IL-6, IL-1β, and GM-CSF) in B16F-10 melanoma cancer cells. Piperine treatment also reduced nuclear translocation of NF-kB subunits p65, p50, and c-Rel, as well as another transcription factors like c-Fos, CREB, and ATF-2. Piperine inhibits NF-κB activation by slowing down IκBα breakdown and p65 translocation from the cytoplasm towards the nucleus (Chung, 2019). Piperine also has a strong binding affinity for the SARS-CoV-2 spike glycoprotein and the ACE2 receptor. - Piperine therapy successfully suppressed IL-6 production by IL-1β activated fibroblast like synoviocytes (FLS) obtained from rheumatoid arthritis patients. Piperine also blocked throw movement of activator protein-1 into the nucleus, but not NF-κB factor(Buagaew and Poomipark, 2020).
**15. Berberine, Isocolumbin, Magnoflorine and Tinocordiside** 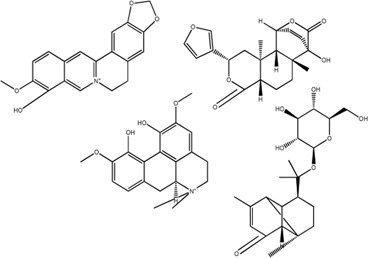	*Tinospora cordifolia* called (Guduchi), *Annona sps* Menispermaceae, Annonaceae family.	-Its wide range of therapeutic qualities, including: Antioxidant, anti-diabetic, anti-arthritic, anti-malarial, immunomodulatory activities, anti-allergic, anti-stress, and anti-inflammatory (Sharma et al., 2012). - Guduchi are components (Alkaloids, aliphatic, glycosides, steroids, and diterpenoid lactones) that are responsible for its therapeutic benefits (Upadhyay et al., 2010)	- **Berberine** modulates the M^pro^ protein activity and consequently suppresses viral multiplication, according to molecular dynamics studies (Chowdhury, 2020). - **Tinocordiside**, greatly reduced the electrostatic contact between ACE2-RBD complexes, resulting in an increase in the complex's flexibility. (Balkrishna et al., 2021). - **Isocolumbin, Berberine, Tinocordiside, and Magnoflorine** had the strongest binding affinity with a main key of SARS-CoV-2 target like surface glycoprotein (6VSB) and receptor binding domain (6MoJ) and RNA polymerase, 6M71) and main protease, 6Y84)] which are responsible for virus attachment to host cells (Sagar and Kumar, 2020). - *T. cordifolia* combined with other medicinal plant called ayurvedic to the SARS-CoV-2 exposed asymptomatic group improves and rejuvenates their immune system. *T. cordifolia* extract has anti-inflammatory characteristics by suppressing proinflammatory cytokines like (TNF-α, IL-6, IL-17 and IL-1β,)/in LPS-stimulated Raw 264.7 macrophages, a neuroinflammatory model in albino rat, and an model arthritic (Sannegowda et al., 2015).	- Guduchi Ghan Vati is being tested in clinical trials to manage and treat COVID-19/CoV-2 (NCT-04480398). *T. cordifolia* extract silver nanoparticles (AgNPs) at a dose of 0.250 mg/mL raised the survival of chikungunya virus-infected cells, indicating the plant's promising applications as an antiviral drug in the form of AgNPs, which can also give a suitable therapeutic for SARS-CoV-2(Sharma et al., 2019).
**16. Glycyrrhizin, 18-β-Glycyrrhetinic acid, Liquiritigenin and Glabridin** 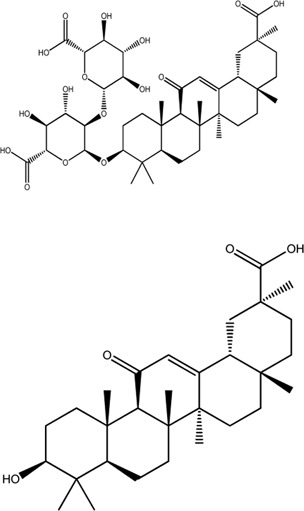 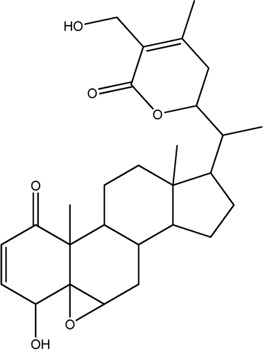	*Glycyrrhiza glabra* (GA), Fabaceae family.	Anti-inflammatory, hepatoprotective, anti-carcinogenic, anti-lung injury and anti-viral properties. .	- **Glycyrrhetinic acid** (GA) and **Glycyrrhizin** (GL), inhibits (11*β*-HSD2, 11*β*-hydroxysteroid dehydrogenase) and stimulates MR in lungs tissues (Murck, 2020). - Glycyrrhetinic acid (**GA**), Liquiritigenin (**L**), and Glabridin (**G**), inhibit the enzyme activities of M^pro^ by forming a strong bond with the active site. However, GA had a higher binding affinity of (-8.0) Kcal/mol than the other two chemicals(Srivastava et al., 2020).. - GA has anti-inflammatory properties due to its targeting of the toll-like receptor (TLR)-4, as well as the ability to block TMPRSS2 and so decrease viral uptake(Murck, 2020). - GA treatment reduced sepsis-induced ALI in mice by lowering oxidative stress, pro-inflammatory reactions, and apoptosis in lung tissue by inhibiting the JNK, and MAPK NF-κB signalling pathways(Zhao et al., 2016). - GL reduced the number of invading overall immune cells, expression of inflammatory cytokines (IL-1, IL-6, and TNFα), neutrophils, and macrophages in BALF and lung parenchyma, compared to the LPS group, by inhibiting the activity CXCR4/CXCR1 activity on neutrophils (Am Lee et al., 2019). [99]. - A recent study found that the glycoside molecule of *G. glabra* binds efficiently to the HMG box protein HMGB1, which is important for virus infection and replication (Bailly and Vergoten, 2020).	- Liquiritigenin and **Glycyrrhizin** inhibits the severity of COVID-CoV-2 patients via working in two stages: it prevents virus entry by decreasing ACE-2 and TMPRSS2expression, and it also reduces pulmonary inflammation individually of ACE-2 [92] (van de Sand et al., 2020). - Clinical trials gov. (**NCT-044241349, NCT-043465887, NCT-03348670, NCT-04487964, NCT-04553705**) found that **glycyrrhizin** had potent a synergistic effect with spironolactone hormone (SP) used to treat COVID-CoV-2 infections (Armanini et al., 2020). - GA could also be regarded the strongest *G. glabra* molecule for fighting SARS-CoV-2 (Sinha et al., 2020). As a result, numerous studies have revealed that, after consulting with an ayurvedic practitioner, *G. glabra* can be used as an immune booster that can help prevent and manage COVID-19 (Sun et al., 2020). - GL & GA could be used as phyto-therapeutic activities to reduce inflammation and lung damage caused by SARS-CoV-2 infection - GL used to patients with ALI reduced inflammation, discomfort, and lung damage by inhibiting the TLR2 signalling pathway (Kong et al., 2019). Furthermore, in an ischemia-reperfusion (I/R) lung damage model, GL (at 200 mg/kg) was found to reduce TLR2-mediated signalling in lung tissue as well as alveolar macrophages (Fei et al., 2017).

Curcumin, piperine, luteolin, phenylnaphthalene, withanolides, anonaine, resveratrol, emodin, anthrarufin, and caffeine are natural immunomodulatory compounds obtained from plant sources known to limit the generation and secretion of inflammatory cytokines/chemokines (Hamed et al., 2019; El-Gengaihi et al., 2020a; Rolta et al., 2021a; Rolta et al., 2021b; Mohammed et al., 2022b; Mohammed et al., 2022c; Rolta et al., 2022). This inhibitory impact is mediated through analysis and planning involved in the generation and release of cytokines and chemokines (e.g., NF-κB, JAK/STAT, and MAPK/ERK). In the combined effect of antiviral medications and other therapeutic drugs now in use, the use of these natural immunosuppressive drugs as adjuvants to reduce the cytokine storms presents a novel, synergistic strategy for the therapies and effective recovery of COVID-19 patients. The effects of luteolin on TNF-induced phosphorylation, nuclear translocation, and DNA binding of NF-κB were significantly decreased. The TNF-induced expression level of the NFKB1 and RELA genes, which encode the two NF-κB subunits (p50 and p65, respectively), was also reduced by luteolin treatment, suggesting that luteolin suppresses pro-inflammatory cytokine production through decreasing NF-κB induction (Kaur et al., 2015).

Although the benefits of these synthetic therapeutic immunomodulatory drugs are undeniable, they are typically coupled with negative side effects. With chloroquine and hydroxychloroquine treatment, cardiomyopathy, neurological, and gastrointestinal side effects have been observed, and because COVID-19 patients are more sensitive due to co-morbidities, there are worries about safety (Bull-Otterson et al., 2020; Colafrancesco et al., 2020; Gevers et al., 2020). Therapies for COVID-19 patients using IL-6 receptor antagonists, such as sarilumab and tocilizumab drugs, which have been linked to endocrinological, hematological, gastrointestinal, and cardiovascular side effects, raise safety concerns (Atal and Fatima, 2020). The side effects of IL-2 inhibitors of cyclosporine and tacrolimus are well-known and can be quite dangerous (Cure et al., 2020). IL-1 blockers, such as anakinra and canakinumab, have a relatively favorable safety profile. However, they can cause elevated liver enzyme levels, myopathy, and moderate leukopenia (Table 2) (Colafrancesco et al., 2020). Anti-TNF therapy side effects have been described infrequently (Caso et al., 2020). Ruxolitinib, a JAK/STAT inhibitor, was delivered to participants with COVID-19 and was shown to cause severe medication responses, causing the study’s early termination (Esposito et al., 2011). With the administration of another oral JAK/STAT inhibitor, baricitinib, there is a risk of severe infections.

Statin treatment in patients with ARDS and sepsis has yielded unsatisfactory results, leading to skepticism about using them as COVID-19 therapy adjuncts. Hypernatremia and dysphagia were observed as side effects in a study of rhACE2 medication for patients with ARDS (Barone et al., 2020). There is a risk of lethal unfavorable thrombotic complications and reactions if MSCs are administered incorrectly, demanding a complete comprehension of the option and application routes of this therapy (Moll et al., 2020).

## 5 Conclusion

COVID/SARS-CoV-2 has sparked widespread alarm and, in a short time, has become a pandemic. Scientists from all over the world are working around the clock to develop efficient medications to combat this unusual viral disease. We provided an overview of the pharmacological targets found in COVID-CoV-2 in this brief analysis. We summarized the single-target and multi-target techniques in depth in addition to the target present. It is conceivable to deduce from the findings of many studies that the multi-target method is preferable to the single-target approach. Although very few studies are available, the multi-target strategy is steadily gaining traction in the field of drug discovery. We also discussed the medications currently in clinical trials and their inhibitory mechanisms near the end of this review. Numerous vaccine studies are receiving greater attention in clinical studies than drug therapy trials. The extensive study offered here may aid in understanding the many pharmacological targets and the multi-target strategy against SARS-CoV-2. The multi-target technique, while promising, has a different risk-benefit profile. Inter-medication has a substantial risk of hazard because unwanted targeting and targeting of host homolog proteins can have negative consequences. Compounds that are big and do not conform to Lipinski’s rule are harmful to the host and have higher drug accumulation and less bioavailability.

As a result, the identification of safe and effective COVID-19 treatments with minimal side effects is critical. Several *in silico* analyses have revealed that natural chemicals derived from plants could be efficient antiviral agents against SARS-CoV-2. Plants produce a staggering array of natural chemicals, many of which have enormous medicinal promise. However, a lack of understanding of natural compound mechanisms of action is a flaw that effectively hinders medical practitioners from accepting plants as phytotherapy. For the prevention and management of COVID-19, the Indian government’s Ministries of AYUSH recently proposed various immunity-boosting methods based on Ayurveda (the oldest healing science, dating back to 5000 years). The WHO recently recommended traditional ancient herbs in the therapies for COVID-19-related health problems that can be used. We have discussed some of the most potent phytotherapeutics present in traditional natural medicines that doctors can use as therapeutic agents to decrease viral infection and toxicity associated with SARS-CoV-2 in this review. We also provided guidance on how to better understand the value of natural plants and how to employ them to strengthen the host’s antiviral defenses.

## Glossary

SARS: severe acute respiratory syndrome
MERS: Middle East respiratory syndrome
CRS: cytokine release syndrome
DIC: diffuse intravascular coagulation
MOF: multiple organ failure
ARDS: abrupt respiratory distress syndrome
Sepsis: early recognition and optimized treatment
DAMPs: damage-associated patterns
PAMPs: pathogen-associated patterns
Tfh cell: T follicular helper cell
IFN- ℽ: interferon-gamma
MCP-1: monocyte chemoattractant protein-1
MIP-1: macrophage inflammatory protein-1
IL-6: interleukin-6
IP-10: interferon-induced protein-10
TNF-α: tumor necrosis factor alfa
MAS: macrophage activation syndrome
ACE-2: angiotensin-converting enzyme-2
TAK: Janus kinase
COPD: chronic obstructive pulmonary disease
ATRA: all trans-retinoic acid
MIS-C: multisystem inflammatory syndrome in children
S-CSS: associated cytokine storm syndrome
NLRP-3: NLR-family pyrin domain containing 3
RBD: receptor binding domain
IAVG: Independent Allocation of Vaccines Group
RAAS: renin–angiotensin–aldosterone system
G-CSF: granulocyte colony-stimulating factor
GM-CSF: granulocyte–macrophage colony-stimulating factor
M-CSF: macrophage colony-stimulating factor
HGF: hepatocyte growth factor
CXCL-8: C-X-C motif chemokine ligand 8
ICU: intensive care unit
RA: rheumatoid arthritis
GVHD: graft-*versus*-host disease
ICPI: intracerebral pathogenicity index
