# Supplementary material for: Fighting cytokine storm and immunomodulatory deficiency: By using natural products therapy up to now
Source: Front Pharmacol. 2023 Apr 12;14:1111329. doi: 10.3389/fphar.2023.1111329 (PMC10134036; doi:10.3389/fphar.2023.1111329)
Supplement: Supplementary file 1 [file Table1.docx]

**Title:** Fighting cytokine storm and immunomodulatory deficiency: By using natural products therapy up to now. ***Review articles***.

**Mona A. Mohammed***

Medicinal and Aromatic Plants Research Department, Pharmaceutical and Drug Industries Research Institute, National Research Centre, Giza, Egypt. Affiliation ID: 60014618.

*Corresponding author e-mail: [monaarafamohammed@yahoo.com](mailto:monaarafamohammed@yahoo.com), [on.ibrahim@nrc.sci.eg](mailto:on.ibrahim@nrc.sci.eg)

**Supplementary Table S1.** **Chemical structure of Natural compound list as Immunosuppressants as Adjuvants in managing COVID-19 to Target the Cytokine Storm.**

| **Natural metabolites, Groups** | **Sources and part was used** | **Therapeutic properties/** | **Mode of Action/ Docking studies** | **Clinical Trials** |
| --- | --- | --- | --- | --- |
| 1. **Curcumin**   [(1E,6E)-1,7-bis(4-hydroxy-3-methoxyphenyl) hepta-1,6-diene-3,5-dione] , Polyphenol group.  **** | *Curcuma longa*, Zingiberaceae family  the rhizomes part are used | Natural antioxidant , Anti-inflammatory, Neuroprotective,  Hepatoprotective , Stop tumour cells, and Immunomodulator (Hewlings and Kalman, 2017, Hamed et al., 2019). | - It used a decrease secretion of proinflammatory cytokines/chemokines such as (IL-1β, IL-6, IL-2, IL-12, TNFα, IFNγ, IP10, CXCL8, MCP1, and MIP1α) in monocyte culture exposed to preeclamptic serum (Rahardjo et al., 2014).  - It blocked chemokines NF/κB pathway by IκBα hydrolysis or stimulating AMPK to damage the NF/κB pathway by focusing on p65 and p50. (Sordillo and Helson, 2015, Shimizu et al., 2019, Liu and Ying, 2020).  - It performs as an immunosuppressant by decreasing IKKβ activation.(Sordillo and Helson, 2015, Shimizu et al., 2019, Liu and Ying, 2020).  Curcumin was suppressed the production of the IL-18/ in LPS-expression murine macrophage such as RAW264.7 cells(Yadav et al., 2015). | - In COVID-19 patients 40 sick clinical from Tabriz University of Medical Sciences' Imam Reza Hospital was conducted in Iran. The nano-curcumin160 mg in capsules daily for fourteen days reduce the levels of hypperinflammatory cytokines: (IL-6 & IL-1β) other hand, had no effect on IL-18 mRNA expression or TNFα levels (Babaei et al., 2020, Valizadeh et al., 2020, Zahedipour et al., 2020). - The clinical study into curcumin's potential as an immunomodulatory adjuvant to lessen the cytokines storms, hyperinflammation, and ARDS associated with SARSCoV-2 infection. (Sordillo and Helson, 2015, Liu and Ying, 2020). |
| 1. **Resveratrol**   5-[(E)-2-(4-hydroxyphenyl) ethenyl]benzene-1,3-diol], phytoalexin polyphenol group.  **** | Grapes, cranberries, blueberries, mulberries, peanuts,  jackfruit, soy, wines. | Natural immunomodulator (Malaguarnera, 2019), antioxidant, antimicrobial(Attia et al., 2021), chemotherapeutic, anti - inflammatory properties, antiviral, anti-aging, and life-prolonging properties. (Bansal et al., 2018). | - It inhibited the secretion of production. (IL-2, MCP1, IL-8, and IFNγ) In EV71-infected RD cells(Zhang et al., 2015) and HTLV-1 cells (Rieder et al., 2012) by splenic lymphocytes and inhibited the production of TNFα, IL-12 and IL-17 by peritoneal macrophages in lung inflammatory cells with a reduction in NF-κB and IκBα expression(Gao et al., 2001). - Resveratrol's **treated in pig's** had ability to reduce IKK activity coincides with its inhibitory effect on the NF-κB pathway therefore influencing humoral immune responses(Fu et al., 2018). - **In a rabbit** model of acute pharyngitis, resveratrol therapy reduced protein expression of IL-1β , IL-18,TNFα, IL-6, MIP2, and p-NF-κB and elevated while suppressing TLR4 and myeloid differentiation primary response protein 88 expression (Zhou et al., 2018). - Phase 2 /Resveratrol’s multimodal antiviral, anti-inflammatory, and antioxidant properties as well as its ability to upregulate ACE_2_ receptors could be helpful in reducing the clinical effects of COVID. | -Resveratrol inhibited the release of inflammatory cytokines and chemokines (GM-CSF and CXCL8) by activated alveolar macrophages from COPD patients, table (4) (Culpitt et al., 2003).  -Resveratrol used as an additional treatment for SARS-CoV-2 infection in order to minimise inflammation by reducing the cytokine storm (Marinella, 2020).  Clinical.Trials.gov: NCT04400890, NCT04799743, NCT04542993 |
| 1. **Gallic** **Acid and polygallic acid**   3,4,5-trihydroxybenzonic acid, phenolic metabolite. | Many fruits as blackberry, blueberry, strawberry, grapes, cashew nut, plumsmango, walnut, hazelnut, and tea. | Natural antioxidants, antimicrobial, antifungal, anticancer, anti-ulcerative colitis and antiinflammatory properties (Zhu et al., 2019). | - Inhibits in TNBS-stimulant, the hyper-inflammatory cytokine ( IL-1/TGF/IL-6/IL-17/IL-12/IL-23/TNFα), chemokines (CCL2 & CCL7) and NF-B pathway (Zhu et al., 2019). - Inhibits the Th2 cells cytokine as (IL-4 & IL-5) but not the Th1 cells cytokines IFNγ in anti-CD3-stimulated/spleen cells, according to (Kato et al., 2001). - ****Block PMA adds A23187-induced IκBα destruction and p65 in NF-κB nuclear dislocation. - In stimulated HMC-1 cells, gallic acid and polygallic acid administration dramatically reduced the release of both of these cytokines (Kim et al., 2006) - Gallic acid could be utilized as an immunosuppression adjuvant to decrease inflammatory cells selectively, so target the cytokine release syndrome seen in SARS-CoV-2 patients without impairing the owner's ability to produce interferon’s. | -In high glucose-induced human monocytes (THP-1 cells), GA treatment reduced NF-κB signaling and reduced proinflammatory cytokines as IL-6 production (Lee et al., 2015)  In human mast cells (HMC-1) was decrease the production of pro-inflammatory cytokine (IL-6 & TNFα). PMA with A23187 were utilized to activate HMC-1 cells, leading to the release of TNFα and IL-6. |
| 1. **Luteolin**   [2-(3,4-dihydroxyphenyl)-5,7-dihydroxychromen-4-one] a naturally occurring flavonoid  **** | Many vegetables (celery, broccoli, parsley, peppers, carrots, and cabbages) as well as in fruits (apple skins), in flowers (chrysanthemum), and others  medicinal herbs contain this compound. | It's a strong antioxidant that stops tumour cells from proliferating and reduces metastasis. It also functions as an anti-proinflammatory and immune function modulator. (Lin et al., 2008). | - Inhibition production of TNFα, IL-1β, IL-6, NF-B, STAT1, in rats with MSU-induced inflammation(Lodhi et al., 2020) and IRF1 suppression (Kao et al., 2011). - luteolin administration on PMA plus A23187-induced HMC-1 cells resulted in considerable reduction of (TNFα, IL-6, CXCL8 & GM-CS), and NF-κB activation suppression (Kang et al., 2010). - A reduce in AP1 transcription factor specific binding activity and block JNK phosphorylation, which had no effect on the LPS-induced increase in NF-κB DNA binding activity or even the LPS-induced degradation of IκBα. - luteolin pretreatment resulted in a dose-dependent reduction in mRNA level expression and the degrade release of IL-6, IL-8, &VEGF in human HaCaT and primary keratinocytes. | -Luteolin has wide antiviral action, block mast cells, and may block COVID/SARS-CoV-2 major protease (3CLpro) (Theoharides, 2020).  - luteolin is a immunomodulatory properties essential natural medication to examine as a therapeutic treatment that could be further investigated as an adjuvant to reduce the cytokines storms seen in COVID-19 and potentially decrease SARSCoV-2 infection. More clinical trials in this area are needed to determine the safety, effectiveness and dose for COVID-19 therapy.   - Luteolin suppressed NF-κB activity in human monocytes in hyperglycemic conditions, resulting in a considerable reduction in the release of IL-6 and TNFα. Luteolin significantly inhibited the generation of IL-1β, IL-6, TNFα, and IFNγ in human whole blood treated with LPS(Ribeiro et al., 2015). - Clinical.Trials.gov: NCT05311852 |
| 1. **Quercetin**   Flavonoid as [2-(3,4-dihydroxyphenyl)3,5,7-trihydroxychromen-4-one].  1-Prophylaxis: 500 mg/daily. Treatment: 1000 mg/daily   - 1. /daily for 1st week; 400 mg/daily for 2nd week Phase 3  1. ****Two tablet twice a day Phase 1, 4-400 mg/daily Phase 3 | many plants including broccoli, red onions, eggplant, potatoes and green leafy vegetables including celery, lettuce; fruits including apples, citrus fruits, red grapes, tomatoes; berries include cranberries and raspberries. | Quercetin an excellent candidate for dealing with situations in which oxidative stress, inflammation, Rheumatoid arthritis, hypercholesterolemia, cardiovascular disease, anti-cancer (Li et al., 2016) and the immune system are involved. | - Reduce the secretion of hyper-inflammatory cytokine such as (TNFα, IL-6, G-CSF, GM-CSF, and VEGF), as well as chemokine (IP-10 and MCP-1), and increase the level of anti-inflammatory cytokine IL- 27 (Mehrbod et al., 2018). - In cultured blood peripheral mononuclear cells, it was also discovered to induce T-helper cells to give IFNγ and to down regulate Th2-cells derived IL-4 (Colunga Biancatelli et al., 2020). - Quercetin therapy considerably inhibited carrageenin-induced IL-1β production. - Inhibit the secretion of inflammatory cytokine is due to its inhibition of the activity of ERK & p38 MAP kinase, as well as the NF-κB/IκB signalling pathways (Tang et al., 2019). - Quercetin inhibited the activation of phosphorylated ERK kinase and p38 MAP kinase in LPS-stimulated RAW 264.7 cells. There was no discernible effect on JNK MAP kinase(Tang et al., 2019). | - Treatment with quercetin reduced the levels of IL-1β, TNFα, and IL-10 in patients with coronary artery disease, according to another study. This was related to transcriptional factor NF-κB activity being reduced (Chekalina et al., 2018). - Quercetin treatment (Oral supplementary) is comparatively safe, having no severe side effects; the only side effects detected in two out of thirty patients in a study were headache and brief peripheral paresthesia. - The use of quercetin as an additive to improve the immune response by promoting IFNs production and changing levels of pro-inflammatory cytokines could be a useful addition to current COVID-19 therapies. (Colunga Biancatelli et al., 2020). - All Clinical trial studied/ 15 number tell now: Clinical.Trials.gov: NCT04377789, NCT04861298, NCT04853199, NCT04578158 |
| 1. **Cinnamaldehyde and Cinnamic acid.**   [(E)-3-phenylprop-2-enal]  **** | *Cinnamomum verum*, *Cinnamomum burmanii Cinnamomum zeylanicum, Cinnamomum cassia*, *Cinnamomum loureirii, Cinnamomum tamala*, &*Cinnamomum cordatum.* | anti-gastric ulcer, antioxidants, anti-inflammatory, anti-microbial, anti-yeast(Dorri et al., 2018), anti-platelet, and hepatoprotective properties that could help manage COVID-19 infection (Gautam et al., 2020).  Cinnamon can thus be used as an antidote to both natural and chemical toxins. | - Cinnamon inhibits the release of IL-1β, IL-6, TNF-α, and nitric oxide (NO) molecules (Ho et al., 2013). - Reduced LPS-induced IL-8 secretion in THP-1 monocytes through altering the TLR-2 and TLR-4 signalling pathways(Schink et al., 2018). | - They have the ability to bind HSPA5 substrate binding domain *β* (SBD- *β*) with a binding energy of -6.25 *±*  1.10, potentially interfering with COVID/SARS-CoV-2 recognition and binding (Elfiky, 2021). |
| 1. **Pavetannin C1 and Tenufolin**   **Bioflavonoids groups**  **** | *Cinnamomum zeylanicum bark* | **:** antioxidants, anti-inflammatory (Dorri et al., 2018). | -Tenufolin and Pavetannin C1, have good affinity with the main proteases a binding energy as ( -8.8 and -7.3) and a high affinity with spike proteins of SARS-CoV-2 a binding energy as (-8.7 and -11.1), respectively (Prasanth et al., 2020, Sasidharan et al., 2022).  . | Clinical trials have demonstrated its efficacy (Sasidharan et al., 2022). |
| 1. **Allicin**   [3-prop-2-enylsulfinylsulfanylprop-1-ene], called thiosulphate Group. | Garlic oil is the component that gives garlic its unique smell and flavour (*Allium sativum*). Allicin can also be found in the field onion (*Allium cepa*) and another Alliaceae species. (Borlinghaus et al., 2014).  **** | antioxidant, antimicrobial, antiviral, antiinflammatory, antitumor and antidiabetic properties (El-Saber Batiha et al., 2020) | - Allicin inhibited TNF-α induced production of (IL-1β, CXCL-8, IP-10, and MIG in HT-29), NF-κB pathway suppresses the breakdown of IκB, resulting in a decrease in cytokine production (Lang et al., 2004). - Allicin therapy increased defense and survival in BALB/c mice after Plasmodium yoelii infection due to an increase in IFNγ production and proliferation of CD4^+^T-cells. - Allicin supplementation combined with tamoxifen treatment resulted in a significant drop in TNF-α levels in Ehrlich ascites carcinoma (EAC) cells, demonstrating its therapeutic effects as an adjuvant (Arreola et al., 2015). | - There were no side effects recorded in clinical trials examining the safety of allicin therapy and pharmacokinetics (with antiviral drugs in use) in COVID-19(Sharifi-Rad et al., 2019). - In a study with cultivated human umbilical vein endothelial cells, allicin reduced TNFα, CXCL8, NFB activity levels and LPS-induced inflammatory reactions (HUVECs) (Zhang et al., 2017). - Inhibits NF-κB signaling for decreased cytokines secretion, improve protection by raising IFNγ production for stronger improved antiviral defense, and promote CD4^+^ T-cell expansion therefore targeting lymphocytopenia, allicin is a highly promising immunomodulatory adjuvant which can be utilized in concert with antiviral medication in COVID-19 patients. |
| 1. **Eugenol compound**   ****2-methoxy-4-prop-2-enylphenol | Clove buds (Syzygium aromaticum), tulsi leaves, cinnamon bark and leaves, pepper, turmeric, ginger, and natural herbs such as oregano, mace, basil, thyme, bay, marjoram, and nutmeg (Khalil and Rahman, 2017). | Anticancer, antioxidant, analgesic, antimicrobial, anticonvulsant, and antimicrobial properties, anti-inflammatory and antiviral activities, as well as being an immunomodulatory drug (Pramod et al., 2010, Dibazar et al., 2015). | - Eugenol inhibited the production of IL-6 and IL-10, IL-4 and IL-5, as well as NF-κB pathways, and thereby OVA-induced eosinophilia was guarded from lung cells study (Bachiega et al., 2012). - Eugenol also inhibited TNFα and IL-1β production as well as the signaling NF-κB pathways (ERK1/2 & p38- MAPK /in LPS-stimulated macrophages) (Barboza et al., 2018). - Eugenol enervated the inflammatory response in pig intestinal epithelial cells by dramatically lowering both CXCL8 and TNFα mRNA levels in an LPS-induced inflammatory model (Hui et al., 2020). | **-** It may be a suitable natural immunosuppressant that can be utilized in combination with antiviral medicines to suppress COVID-19's hyper-cytokinemia and hyper-inflammation. |
| 1. **6-shogaol and 6-gingerol compounds**   ****[**6-gingerol,** (5S)-5-hydroxy-1-(4-hydroxy-3-methoxyphenyl)decan-3-one], [**6-shogaol,** (*E*)-1-(4-hydroxy-3-methoxyphenyl)dec-4-en-3-one] | *Zingiber officinale* rhizomes belongs to Zingiberaceae family  and traditional medicine from ancient times. | antioxidant, anti-metastatic, anti-inflammatory, anti-angiogenic, analgesic, anti-diabetic, and antipyretic properties. Furthermore, gingerol is reported to have anti-allergic, neuroprotective, immunomodulatory, and anti-carcinogenic activities. (Sharifi-Rad et al., 2017). | - In LPS-stimulated macrophages, 6-gingerol was decrease e production of cytokines IL-1β, IL-12, & TNFα and chemokine CXCL8 & COX2 without interfering with their antigen presentation function and AMPK activation(Tripathi et al., 2007, Sheng et al., 2020). - Inhibits NF-κB pathway being down-regulated as a result of an increase in phosphorylated IKBα and p65 downregulation (Saha et al., 2016). | - An *in silico* analysis found that 6-shogaol and 6-gingerol compounds have a high affinity of S-spike protein in SARS-CoV2 and ACE-2 receptor, suggesting that they could help reduce viral load and SARS-CoV-2 shedding in the nasal passage (Haridas et al., 2021). - After being used in traditional antiviral medication, 6-gingerol is a potent natural immunotherapeutic drug that could help control the cytokine storm seen in COVID-19. |
| 1. **Melatonin**   N-acetyl-5- methoxytryptamine,  acetamides groups. | *Curcuma aeruginosa*, *Piper nigrum*, *Oryza sativa*, *Zea mays*, *Brassica hirta*, *Apium graveolens*, *Helianthus annuus*, *Foeniculum* *vulgare*, *Prunus amygdalus*, *Coriandrum sativum*, *Pimpinella anisum*, and Coffea arabica L. It has been found in several plant species from the Brassicaceae, Poaceae, Vitaceae, Rosaceae, and Apiaceae, families (Nawaz et al., 2016).  **** | Antioxidant, anti-inflammatory, anti-viral activity, and anti-pro-inflammatory cytokines (Salehi et al., 2019). | - Melatonin inhibited NF-κB/STAT1/3STAT/GAS/, a major transcription factor as inflammatory cytokines (Min et al., 2012). - Melatonin decreases in serum levels of IL-6, TNFα, and IL-1β (Zhang et al., 2020). - In a study of male C57BL/6 mice with cadmium-induced liver injury, melatonin treatment was discovered decrease response inflammatory cytokine secretion and block NLRP3 cells inflammasome activation (Cao et al., 2017). - Melatonin therapy effectively decreased the LPS- effected generation of many chemokine MCP1, CCL9, and CCL5 mRNA expression in BV2 murine microglial cells in another investigation. | -Melatonin strongly inhibited IL-8 production in acrolein-stimulated human lung fibroblasts. (Kim et al., 2012).   - According to a recent mechanistic investigation, melatonin treatment can effectively reduce the cytokines storms in COVID-19 by reversing aerobic glycolysis in system immune cells (Reiter et al., 2020).   These findings support the use of melatonin as an immunosuppressive supplement to reduce the cytokines storms syndrome seen in COVID-19. (Zhang et al., 2020).   - All Clinical trial studied/ 11 number tell now: Clinical.Trials.gov: NCT04474483,   NCT04409522, NCT04568863, NCT04530539, NCT04353128. |
| 1. **Morphine** **and Codeine compounds**   An opiate alkaloid groups  **[morphine**, (4R,4aR,7S,7aR,12bS)-3-methyl-2,4,4a,7,7a,13-hexahydro-1H-4,12 methanobenzofuro[3,2-e]isoquinoline-7,9-diol)**]** binds to a specific the μ,δ,κ opiate receptors involved in modulating various activity of the brain and **[Codeine**,(4R,4aR,7S,7aR,12bS)-9-methoxy-3-methyl-2,4,4a,7,7a,13-hexahydro-1H-4,12-methanobenzofuro[3,2-e]isoquinolin-7ol)].  **** | *Papaver somniferum* | Anti-diarrheal, analgesic, and antitussive effects.  Morphine and codeine are thus potential immunosuppressant adjuvants for reducing the cytokine storm seen in COVID-19 | - Morphine has decrease secretion of IFNγ, IL-6, IL-12, IL-1β, MCP 1, and TNFα in bronchoalveolar lavage fluids and pulmonary tissue of CB6F1 mice (Fukada et al., 2012) - In lung resident cells, morphine therapy suppressed the transcription factor NF-κB. A-stimulated splenocytes extracted from male Swiss mice, codeine treatment significantly reduced the generation of IL-2. - Docking tests demonstrated that morphine and codeine bind to ACE2 with a high affinity. This could theoretically limit cytokine release mediated by receptors. (Madera-Salcedo et al., 2011). | - Morphine's immunosuppressive action in relation to cytokines have been proven in clinical practice for over a century (Dinda et al., 2005). - **Ho**wever, more research is needed to investigate whether morphine or codeine should be used to treat COVID-19, because the time of morphine treatment affects cytokines secretion; Codeine has also been discovered to promote the synthesis of cytokines and chemokines by mast cells in vitro, and late treatment improves cytokine output. (Fukada et al., 2016). - All Clinical trial studied/ 2 number tell now: Clinical.Trials.gov: NCT04522037, NCT05381883. |
| 1. **Nicotine**   3-(1-methylpyrrolidin-2-yl) pyridine, Alkaloid groups | *Nicotiana tabacum,* Crassulaceae, Asclepiadaceae, and Solanaceae families. | It's a neuropsychiatric drug, an immunomodulator, as well as a drug for the peripheral nervous system. (Peter et al., 2021). | - The main mechanism of action involves the stimulation of a7-nAChRs onto inflammatory cells such as macrophages and neutrophils, which results in the suppression of NF-κB activation and, as a result, the release of pro-inflammatory cytokines and chemokines by these cells. - In a study with PBMC induced by HT-29 colon cancer cells, nicotine inhibited the production of (TNFα, IL-1β, IFNγ, and IL-2) but had no effect on the activity of IL-6. Nicotine therapy in PBMC driven by RKO colon cancer cells showed no significant effect on cytokine production (Djaldetti and Bessler, 2017). - **** Nicotine has been shown in studies to decrease the secretion of IL-1, IL-6, IL-12, INF, MIP1, and TNF. Nicotine decreases the phosphorylation of I-B, which reduces the transcriptional activity of NF-B (Piao et al., 2009).. | - It is used to treat ulcerative colitis in the hospital to reduce inflammation. Nicotine inhibits inflammatory cytokines by interacting through the cholinergic anti-inflammatory system through the a7-nicotinic acetylcholine receptor, as it is a cholinergic inhibitor (a7-nAChRs). IL-1β, and IL-6, TNF production are all inhibited by nicotine (Gonzalez-Rubio et al., 2020) - In human macrophages and splenocytes, nicotine reduces NF-κB and TNFα release caused by the LPS system. This inhibitory activity is related to nicotine's ability to stimulate JAK2 and STAT3 in macrophages, which is controlled through tristetrapolin (TTP) expression [172]. - Nicotine is also a widely available and well-recognized therapy. As a result, it is suggested that nicotine is a good adjuvant that can reduce the cytokine storm in COVID-19 and, in the short term, prevent growing mortality (Piao et al., 2009) - Clinical.Trials.gov: NCT04598594 |
| 1. **Piperine**   (2*E*,4*E*)-5-(1,3-benzodioxol-5-yl)-1-piperidin-1-ylpenta-2,4-dien-1-one,  alkaloid **** group | *Piper nigrum* L., Piperaceae species | Antibacterial, antioxidant, anti-metastatic, anti-inflammatory, and hepatoprotective properties, immunomodulator, and it renowned for increasing the bioavailability of medicinal drugs (Gorgani et al., 2017). | - In an ovalbumin-induced asthma model, piperine has been found to block the secretion of Th2 cells cytokine as (IL-4 & IL-5) (Kim and Lee, 2009). - Piperine blocks LPS-stimulated expression and secretion of hyperproinflammatory cytokine such as (IL-10, IL-6, IL-1β, & TNFα through inhibit NF-κB stimulation, and MAPK pathways (Dzoyem et al., 2017) ~~(Liang et al., 2016)~~ (Zhai et al., 2016).. - **Docking studies:** As a result, piperine could be a promising therapeutic treatment not only for preventing virus adhesion to host cells, but also for preventing cytokines storms throw inhibiting the activity of the MAPK and NF-κappaB pathways, which leads to the release of pro-inflammatory cytokines (Maurya et al., 2020). | - Piperine therapy resulted in a significant decrease in the concentrations of (TNFα, IL-6, IL-1β, and GM-CSF) in B16F-10 melanoma cancer cells. Piperine treatment also reduced nuclear translocation of NF-kB subunits p65, p50, and c-Rel, as well as another transcription factors like c-Fos, CREB, and ATF-2. Piperine inhibits NF-κB activation by slowing down IκBα breakdown and p65 translocation from the cytoplasm towards the nucleus (Chung, 2019). Piperine also has a strong binding affinity for the SARS-CoV-2 spike glycoprotein and the ACE2 receptor. - Piperine therapy successfully suppressed IL-6 production by IL-1β activated fibroblast like synoviocytes (FLS) obtained from rheumatoid arthritis patients. Piperine also blocked throw movement of activator protein-1 into the nucleus, but not NF-κB factor(Buagaew and Poomipark, 2020). |
| 1. **Berberine, Isocolumbin, Magnoflorine and Tinocordiside** | *Tinospora cordifolia* called (Guduchi), *Annona sps* Menispermaceae, Annonaceae family. | -Its wide range of therapeutic qualities, including: Antioxidant, anti-diabetic, anti-arthritic, anti-malarial, immunomodulatory activities, anti-allergic, anti-stress, and anti-inflammatory (Sharma et al., 2012).  - Guduchi are components (Alkaloids, aliphatic, glycosides, ****steroids, and diterpenoid lactones) that are responsible for its therapeutic benefits (Upadhyay et al., 2010) | - **Berberine** modulates the M^pro^ protein activity and consequently suppresses viral multiplication, according to molecular dynamics studies (Chowdhury, 2020). - **Tinocordiside**, greatly reduced the electrostatic contact between ACE2-RBD complexes, resulting in an increase in the complex's flexibility. (Balkrishna et al., 2021). - **Isocolumbin, Berberine, Tinocordiside, and Magnoflorine** had the strongest binding affinity with a main key of SARS-CoV-2 target like surface glycoprotein (6VSB) and receptor binding domain (6MoJ) and RNA polymerase, 6M71) and main protease, 6Y84)] which are responsible for virus attachment to host cells (Sagar and Kumar, 2020). - *T. cordifolia* combined with other medicinal plant called ayurvedic to the SARS-CoV-2 exposed asymptomatic group improves and rejuvenates their immune system. *T. cordifolia* extract has anti-inflammatory characteristics by suppressing proinflammatory cytokines like (TNF-α, IL-6, IL-17 and IL-1β,)/in LPS-stimulated Raw 264.7 macrophages, a neuroinflammatory model in albino rat, and an model arthritic (Sannegowda et al., 2015). | - Guduchi Ghan Vati is being tested in clinical trials to manage and treat COVID-19/CoV-2 (NCT-04480398). *T. cordifolia* extract silver nanoparticles (AgNPs) at a dose of 0.250 mg/mL raised the survival of chikungunya virus-infected cells, indicating the plant's promising applications as an antiviral drug in the form of AgNPs, which can also give a suitable therapeutic for SARS-CoV-2(Sharma et al., 2019). |
| 1. **Glycyrrhizin, 18-β-Glycyrrhetinic acid, Liquiritigenin and Glabridin**     ****** | *Glycyrrhiza glabra* (GA), Fabaceae family. | Anti-inflammatory, hepatoprotective, anti-carcinogenic, anti-lung injury and anti-viral properties.  . | - **Glycyrrhetinic acid** (GA) and **Glycyrrhizin** (GL), inhibits (11*β*-HSD2, 11*β*-hydroxysteroid dehydrogenase) and stimulates MR in lungs tissues (Murck, 2020). - Glycyrrhetinic acid (**GA**), Liquiritigenin (**L**), and Glabridin (**G**), inhibit the enzyme activities of M^pro^ by forming a strong bond with the active site. However, GA had a higher binding affinity of (-8.0) Kcal/mol than the other two chemicals(Srivastava et al., 2020).. - GA has anti-inflammatory properties due to its targeting of the toll-like receptor (TLR)-4, as well as the ability to block TMPRSS2 and so decrease viral uptake(Murck, 2020). - GA treatment reduced sepsis-induced ALI in mice by lowering oxidative stress, pro-inflammatory reactions, and apoptosis in lung tissue by inhibiting the JNK, and MAPK NF-κB signalling pathways(Zhao et al., 2016). - GL reduced the number of invading overall immune cells, expression of inflammatory cytokines (IL-1, IL-6, and TNFα), neutrophils, and macrophages in BALF and lung parenchyma, compared to the LPS group, by inhibiting the activity CXCR4/CXCR1 activity on neutrophils (Am Lee et al., 2019). [99]. - A recent study found that the glycoside molecule of *G. glabra* binds efficiently to the HMG box protein HMGB1, which is important for virus infection and replication (Bailly and Vergoten, 2020). | - Liquiritigenin and **Glycyrrhizin** inhibits the severity of COVID-CoV-2 patients via working in two stages: it prevents virus entry by decreasing ACE-2 and TMPRSS2expression, and it also reduces pulmonary inflammation individually of ACE-2 [92] (van de Sand et al., 2020). - Clinical trials gov. (**NCT-044241349, NCT-043465887,** **NCT-03348670, NCT-04487964, NCT-04553705**) found that **glycyrrhizin** had potent a synergistic effect with spironolactone hormone (SP) used to treat COVID-CoV-2 infections (Armanini et al., 2020). - GA could also be regarded the strongest *G. glabra* molecule for fighting SARS-CoV-2 (Sinha et al., 2020). As a result, numerous studies have revealed that, after consulting with an ayurvedic practitioner, *G. glabra* can be used as an immune booster that can help prevent and manage COVID-19 (Sun et al., 2020). - GL & GA could be used as phyto-therapeutic activities to reduce inflammation and lung damage caused by SARS-CoV-2 infection - GL used to patients with ALI reduced inflammation, discomfort, and lung damage by inhibiting the TLR2 signalling pathway (Kong et al., 2019). Furthermore, in an ischemia-reperfusion (I/R) lung damage model, GL (at 200 mg/kg) was found to reduce TLR2-mediated signalling in lung tissue as well as alveolar macrophages (Fei et al., 2017). |
| 1. **Withaferin-A**   a withanolide, as 27-hydroxy steroid group.   | - *Withania somnifera, Physalis peruviana* Solanaceae family | - Anti-Tumorigenic agent, anti-hepato-renal fibrosis(Khalaf-Allah et al., 2016) and anti-*H pylori*[207]. | - *In silico* revealed, withaferin-A had a high affinity with the M^pro^ (-11.2421 kcal/mol) and the RNA Dependent RNA Polymerases enzyme (-9.271 kcal/mol) enzymes (Pandit and Latha, 2020). In the instance of COVID-CoV, papain-like proteases (PL^pro^) have been found to induce oxidative stress by promoting ROS generation and TGF-1 signalling, which leads to lung fibrosis (Kashyap et al., 2020). - **Withaferin A** has the ability to decrease the formation of oxidative stress caused by viral infection (Mandlik and Namdeo, 2021). - **Withaferin-A** is modulating the NLRP-3 inflammasome and NF-kB activation in a dose-dependent manner, therapies inhibited the excitation of the nucleotide-binding domain leucin rich repeat (NLRP3) inflammasome, which also inhibited IL-1β secretion in LPS-triggered macrophages and also prevented Helicobacter pylori-triggered IL-1β generation from dendritic cells [207]. - **Withaferin-A**; LPS-induced lung injury was also reduced by limiting neutrophil infiltration into the lungs, which was followed by a decrease in inflammatory cytokine such as (IL-6& TNF-α). | - Withaferin-A has antiviral and immunomodulatory effects, and has been used to treat PBMCs from HIV patients. - Withaferin-A suppressed CD38 expression in HIV-positive CD8^+^ T cells. CD38 is a CD8^+^ T cell marker that can be utilized as a biomarker to detect HIV progression (Maurya et al., 2019). |
| 1. **Withanoside**-**V, Withanoside-D, Withanoside-G, Withanoside-M, Withanolide-A and Somniferine**    | - *Withania somnifera, Physalis peruviana,* Solanaceae family. - It also is called "Sattvic Kapha Rasayana," and it's a popular Ayurvedic medicine herb. | Antiarthritic, hepatoprotective(El-Gengaihi et al., 2013) , antimicrobial, and anti-tumorigenic agent (Chen et al., 2011) | - **Withanolide-D** and **withanolide-G** had stronger PL^pro^ binding interaction, while **withanolide-M** had the best 3CL^pro^ and spike protein binding affinity. - **Withanolide Q** was shown to affect a greater number of proteins than all of the other withanolides. - **Withanolide** inhibit secretion triggered by inflammation of (TNFα, & IL-1β), triggered (COX-II) & (iNOS). - **Withanolide** block NF-kB, P38, and MAPKs signalling pathways, the aqueous extract of *W. somnifera* reduced the inflammation condition by decreasing the generation of inflammatory cytokines such as IL-1β, IL-6, and TNFα (Gupta and Kaur, 2019). - These studies have led to the conclusion that *W. somnifera* and its physiologically active bioactive constituents can reduce inflammation and oxidative stress linked with a set of inflammatory syndromes, such as viral diseases like COVID-19. | - By altering the electrostatic contacts between the COVID-CoV-2 S-protein receptor binding domain (RBD) and the ACE-2 receptor, W. somnifera can decrease the interconnections (Balkrishna et al., 2020). - Proteases have been discovered to be the most common target for stopping viral replication. The main protease is one of the best targets for coronavirus (M^pro^). M^pro^ cleaves polyproteins needed for SARS-CoV-2 replication and transcription, hence inhibiting M^pro^ will stop the virus from multiplying. Shree et al., (Shree et al., 2020) found that two ashwagandha components, **Withanoside V** (10.321 kcal/mol) and **Somniferine** (9.621 kcal/mol), have a significant affinity for SARS-CoV-2 M^pro^ (mainly protease: a crucial for COVID-19). - Withanoside V is a powerful inhibitor of M^pro^, which is implicated in SARS-CoV-2 replication (Tripathi et al., 2021). - SARS-CoV-2 infects human cells by detecting the Glucose-Regulated Protein 78 (GRP78) receptor throughout its substrates binding domain, in additional to the ACE-2 receptor (SBD). **Withanolide A**, surprisingly, has a greater binding affinity for GRP78 (-8.71 kcal/mol**).** |
| 1. **Andrographolide**   Andrographis is a labdane diterpenoid   | *Andrographis paniculata*, Acanthaceae family | Anti-allergic, hepatoprotective activity, anti-platelet, proantii-inflammatory, , antineoplastic, and anti-HIV (Jayakumar et al., 2013). | - Andrographolie is a strong immunomodulator that has been shown to increase immunological response, control NK cell and cytokine production, and stimulate the formation of cytotoxic T-lymphocytes(Varma et al., 2011). - In LPS/IL-4 activated murine macrophages (RAW264.7 cells), andrographolide effectively reduced the levels of proinflammatory cytokine such as IL-1β, TNFα, IL-12, IL-6, & IL-18 in a dose-response curve (Wang et al., 2010). - Andrographolide inhibited the LPS induced NF-κB and MAPK pathways, inflammatory cytokines production is reduced. - Andrographolide suppresses IL-1β, TNFα, (PGE-2) prostaglandin E2, (NOX-2) NADPH oxidase-2, and (iNOS) inducible nitric oxide synthase in hypoxic brain areas after pMCAO activation (Lu et al., 2019). - Andrographolide lowered the amounts of phosphorylated of p65 and IkB, while in the MAPK pathway; it decreased the values of (p-JNK, p-ERK1/2, and p-p38) (Li et al., 2017). TNF-α/NF-κB and TLR4/NF-B signaling pathways were found to be inhibited by andrographolide derivatives, which inhibited of the NF-κB, p65 and IκB, then lowering serum pro-inflammatory cytokines and chemokines (Nie et al., 2017). - Andrographolide reduced mouse cortical chemokine amount from the (CCL-2, CCL-5) and (CXCL-1, CXCL-2, CXCL-10) as subfamilies considerably in a study. In astrocytes, andrographolide inhibited LPS induced chemokines as (CCL-2, CCL-5, CXCL-1, CXCL-5, CX3CL-1) and TNFα (Wong et al., 2016). - Andrographolide's dual benefits as an immunosuppressive agent that reduces aberrant cytokines/chemokines productivity as well as a potential inhibitor of SARS CoV-2 by targeting the mainly protease make it a promising natural bioactive drug for COVID-19 therapy (Banerjee et al., 2020). | - Andrographolide has showed promise in lowering brain atrophy and disability progression in those with non-progressive MS, as well as a favorable safety profile. With an anti-inflammatory and neuroprotective mechanism of action, more research is needed to validate andrographolide's efficacy and safety in broader populations, as well as to evaluate its use in combination with other highly active disease modifying treatments( NCT-05019326) (Ciampi et al., 2020). - Andrographolide is a strong inhibitor of primary protease SARS-CoV-2. - Andrographolide is a safe substance that does not interfere with the metabolism of other medicines. (Enmozhi et al., 2021). - Clinical.Trials.gov: **NCT05019326, NCT04847518** |
| 1. **Colchicine**   N-[(7S)-1,2,3,10-tetramethoxy-9-oxo-6,7-dihydro-5H-benzo[a]heptalen-7-yl]acetamide according alkaloids group.   | *Colchicum autumnale,* Colchicaceae family | Anti-fibrotic activities, gouty arthritis, Familial Mediterranean Fever, osteoarthritis, pericarditis and atherosclerosis. | - Colchicine inhibited pyrin and NLRP3 inflammasome activation and is currently being studied for COVID-19 treatment (Deftereos et al., 2020, Nabavi et al., 2023). - A single-center cohort research indicated that patients taken colchicine had a greater rate of survival than those treated with standard therapy after twenty one days, with identical adverse effects in both groups, supporting the rationale for using colchicine to treat in COVID-19 (Scarsi et al., 2020, Nabavi et al., 2023). | - Low-dose colchicine provides anti-inflammatory benefits and a good safety profile, according to a randomised clinical trials enrolling 105 case in  COVID-19 sufferers (Deftereos et al., 2020). - It is also advised as a treatment option in patients who have allergies to other drugs or in the event of antiviral drug shortages/unavailability (such as in developing countries) (Piantoni et al., 2020) due to its high availability. - All Clinical trial studied/ 36 numbers tell now: Clinical.Trials.gov: NCT05118737, NCT04392141, NCT04667780, NCT04375202. |
| 1. **Artemi C**:   (Micellar formulation)  Artemisinin (6 mg), Curcumin  (20 mg), Frankincense (15 mg), and  Vitamin C (60 mg) | **-Mixed natural compounds** | - Anti viral | - In Phase II/ Diminish IL-6 and TNF-α levels (Hellou et al., 2022) | - Clinical investigation into the effectiveness of ArtemiC oral spray in reducing symptoms in hospitalised COVID-19 patients. Treatment with ArtemiC was linked to clinical improvement, increased SpO2 levels, and a reduction in fever duration. - ArtemiC prevented deterioration, potentially by reducing the COVID-19 cytokine storm, and hold significant promise for COVID-19 patients, especially those with concomitant conditions. - Clinical.Trials.gov: NCT04382040, NCT04802382. - ICTRP: CTRI202102031520 |
| 1. ***Nigella sativa*** (**NS**)   (Black Cumin)  Black seed (500 mg) and (Cumin seed powder (1 gm), Main compounds Thymoquinone 35-40%  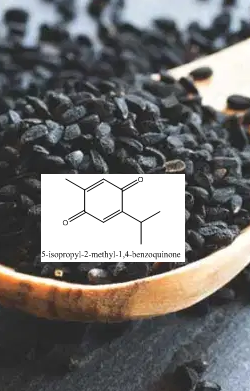 | **Ranunculaceae** | Antihypertensive, liver tonics, diuretics, digestive, anti-diarrheal, appetite stimulant, analgesics, anti-bacterial and in skin disorders. | - After CoV infection, NS extract treatments (Phase II) affected IL-8 secretion, TRP gene expression, and viral load, although the NS extract treatment caused the greatest difference in virus burden. - The best option for a potential therapeutic molecule in our possession is thus NS extracts (Koshak et al., 2021). | - For patients who had a mild COVID-19 infection, NS supplementation was linked to a quicker recovery of symptoms than only standard therapy. Studies that are placebo-controlled and double-blinded are needed to further investigate these potential therapeutic advantages (Koshak et al., 2021). - All Clinical.Trials.gov. studied as 8 trial number up to now: NCT04553705, NCT04401202, NCT04347382. |
| 1. **Previfenon®**   Epigallocatechin-3-Gallate  (EGCG) (250 mg) **, three times per day.**   | **Green tea, Theaceae family** | **chemoprophylaxis as SARS-CoV-2.** | - Phase II/ Exhibits anti-viral chemoprophylaxis of COVID-19 (Chourasia et al., 2021) - According to mechanistic investigations, EGCG prevented infection at the entrance stage by preventing the viral spikes' (RBD) from attaching to the host cells' angiotensin-converting enzyme 2 (ACE2) receptor (Liu et al., 2021) | - Epigallocatechin gallate (EGCG), a key component of green tea beverage (GTB), was very successful in preventing the spread of live SARS-CoV-2 and human coronavirus (HCoV OC43). - GTB or EGCG effectively prevented infection of the pseudoviruses with spikes of the novel types (UK-B.1.1.7, SA-B.1.351, and CA-B.1.429). - EGCG was the most effective against the viruses of the four active green tea catechins at noncytotoxic dosages. When the viruses or the cells were pre-incubated with EGCG before the infection, the maximum inhibitory activity was seen (Liu et al., 2021). - Clinical.Trials.gov: NCT04446065 |
| 1. **Guduchi Ghan Vati**  - An Ayuvedic classical preparation + aqueous extract of *Tinospora cordifolia* **(**Giloin, Giloinin and Gilosterol, Tinosporin along with Berberine**)** - A rich source of terpenes, and alkaloids, diterpenoid lactones, glycosides, steroids, sesquiterpenoid, phenolics, aliphatic compounds and polysaccharides (Kumar et al., 2020)**.** | **Mixed plants** | anti-arthritic, antioxidant, , anti-leprotic, antimalarial, anti-diabetic anti-allergic,, anti-pyretic, antispasmodic, anti-inflammatory, anti-stresshepato-protective, immuno-modulatory and anti-neoplastic activities(Kumar et al., 2020). | - Immunomodulatory potential. - Guduchi was attributed to its capacity to reduce SARS-CoV-2 replication (Thakar et al., 2022). - The major protease present in coronaviruses, 3C-like protease (3CLpro), is directly inhibited by T. cordifolia when it comes to combating SARS-CoV-2. In the xenotrans plant model of humanised zebrafish, Guduchi also restored the illness phenotype caused by the SARS-CoV-2 viral spike-protein (Thakar et al., 2022). | -A promising role of Guduchi Ghan Vati in terms of virologic cure with no side effects.  Guduchi as potent therapeutic agents for treating ailments like Jwara (fever), Vishama Jwara, Swasa, Kasa, and Sotha, among others. Guduchi Ghana Vati, a tablet made from a concentrated aqueous extract of the Guduchi stem, is the most popular form of this herb **(Kumar et al., 2020)**.   - Clinical.Trials.gov: NCT04480398, NCT04542876. |
| 1. ***Gargles* (Mouthwash**) Neem  - ArtemiC, Citrox (a bioflavonoid), cetylpyridinium chloride, chlorhexidine, chlorine dioxide, essential oils, hydrogen peroxide, hypertonic saline, Kerecis spray (omega 3 viruxide - containing neem oil and St John's wort), and neem extract (Khan et al., 2020).   . | **Mixed plants** | - Antiviral gargles could be used by dentist and their auxiliaries as prophylaxis. Anti-viral (Khan et al., 2020). | - Gargling diminished the COVID-19 colonies that were present in the mouth, and it is likely that this reduced the viral infection (Khan et al., 2020). | - Through surface debridement, the viral can be reduced, which may help the immune system respond effectively and improve the patients' general symptoms (Khan et al., 2020).   All Clinical trial studied/ 43 numbers tell now: Clinical.Trials.gov: NCT04341688, NCT04352959, NCT04894409 |
| 1. **Omega 3 Viruxide**   Neem oil (hexadecanoic acid (52.2-72.3%))(Kurose and Yatagai, 2005)& Wort oi sesquiterpene hydrocarbons (77.1%)(Sajjadi et al., 2015) | **Mixed natural oils**: *Azadirachta indica* (Meliaceae), and *Hypericum* (Hypericaceae) | Antidepressive, Wound Spray | -No finish yet  -Reduce symptoms associated with COVID- 19 infection | Clinical.Trials.gov  NCT04357990 |
| 1. **Traditional Chinese medicine** (TCM)   Contains (Alkaloids, Polyphenols, and Terpenoids(Meng et al., 2009).  TCM used in China for more than 2,000 years, and for the past 200 years it used in natural therapy.  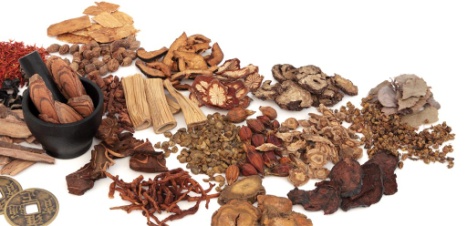  https://thisquarterly.sg/food/traditional-chinese-medicine-an-introduction/ | **Mixed plant groups** | antioxidants, antimicrobials , and opiate addiction treatment (Al-Kuraishy et al., 2022) | -Phase III/ To evaluate the safety and efficacy of TCM  as an adjuvant for the patients with SARS-CoV-2/IgG  COVID-19 | -TCM is useful when treating COVID-19 in reducing the period of positive viral nucleic acid, delaying the onset of symptoms and the improvement to severe disease, and preventing multi-organ injury; and relieving the typical symptoms of fever, cough, fatigue, dry throat, sore throat, sputum production, shortness of breath, myalgia, and diarrhoea.  - Enhancing lung characteristics, such as oxygenation index, CT imaging, lung damage, and absorption of inflammatory substances;  - Controlling on the D-dimer level, which measures thrombosis, as well as inflammatory and immune response measurements such as the WBC, lymphocyte, CD4+ T and CD8+ T counts, as well as the levels of CRP, IL-6, TNF-, and ESR.  - All Clinical trial studied/19 number tell now; Clinical.Trials.gov: NCT04323332, NCT04668222, NCT04492501 |
| 1. **Ayurveda**   Contains (Ginger/Turmeric/Honey/Lemon)  7th August 2020, The AYUSH Ministries of the Indian Government have proposed a number of immunity-boosting techniques based on Ayurveda (the oldest healing science, dating back 5000 years). | **Mixed natural plants** | anti-inflammatory and anti-dyspneic characteristics that reverse inflammation and lessen fibrosis. | - Ayurveda self-management for flu like symptoms during the COVID-19 outbreak | - **Ayurveda** is very helpful for controlling the downward deposition of fibrin. Influenza is inhibited by **Ayurveda** and controlling the innate immune response, a virus can replicate and cause lung immunopathology. - All trial Clinical studied/ 9 number tell now; Clinical.Trials.gov: NCT04345549 |
| 1. **Cannabidiol**   cannabinoids groups  Sublingual oil drops: 3 times a day.   | *Cannabis* plants, Also cannabinoids can be in rhododendron, licorice, liverwort, and Echinacea. | Anticancer, chemotherapy-induced nausea and anti-vomiting, anti epilepsy, anti insomnia, and anti anxiety symptoms(National Academies of Sciences and Medicine, 2017)**.** | - Phase 1/ Phase 2/ Phase 3/ reduces the level of stress, modify inflammatory parameters, such as cytokines, measured from the serum of professionals in the care of patients with COVID-19. | - Cannabidiol reduced coronavirus replication and virus induced cytokine release. - Cannabidiol inhibited Remdesivir hydrolysis - The potential Drug-drug interactions between Cannabidiol and the COVID-19 therapeutic agent Remdesivir should be appreciated by clinicians using the antiviral. - FDA approved clinical trial to treat lung inflammation induced by COVID-19 - All Clinical trial studied/8 number tell now as: NCT04686539, NCT04731116. |
| 1. **Curcumin with Sinacurcumin®**   1- 500 mg curcumin plus 5 mg piperine twice a daily for 2 weeks  2- Sinacurcumin® soft gel: 40 mg; 4 soft gels daily for 2 week | **Mixed natural compounds** |  | - Curcumin can decrease excretion of wide range of cytokines which have critical role in many severe and chronic disease (IL-1, 2, 6, 8, 10, 11, 12, & 17, TNFα, interferon-γ, MCP1, MIP1α, nuclear factor kappa-light-chain enhancer of activated B-cells | - Curcumin affected the pain and quality of life considerably curcumin had a similar performance to sodium diclofenac patients. a combination of curcumin and sodium diclofenac significantly improves the intensity of pain in patients - All clinical trial gov as: (10 studies) NCT03715140 - And IRCT as (3 studies): IRCT20121216011763N46, IRCT20121216011763N52 IRCT20200408046990N1. |
| **31. Combination Product**  Hydroxychloroquine/Azithromycine  **Combination Product:** (4 Plants/Azythromycin) Quinquina-Stevia/Azythromycin/ Cinchona | **Mixed plants**  *Cinchona officinalis,* Rubiaceae family*, Stevia Rebaudiana* | **Antifever**, anti**-malaria and anti-influenza**(Seigneuric et al., 2008), nti-hyperglycemia,anti-oxidative, hypotensive, nephro-protective, hepato protective, antibacterial and antifungal(Ahmad et al., 2020) | - Phase II/ Its immunomodulatory properties may help to limit the unchecked immunological response to SARS-CoV-2. - Less inflammatory cytokine is produced, which minimises tissue and endothelium damage and stops the start and development of autoimmune inflammation (Ortega-Peña and González-Cuevas, 2021). | Clinical.Trials.gov.  NCT04501965 |
| **32. Plant Stanol Esters**  4g of plant | **Mixed natural compounds** |  | - These compounds also beneficially influence the immune system, e.g. increasing vaccine-specific antibody titers. - Plant stanols may reduce the chance that the virus may infect enterocytes by substituting the cholesterol that enterocytes' cell membranes are likely to contain(Plat et al., 2022). | - This impact could have an impact on T cell behaviour. - The impacts of plant stanol ester consumption enhanced regulatory T cell activity, which improved the Th1 response and decreased the Th2 response in asthma patients. - The higher BMIs may benefit more from plant stanol consumption in the weeks before and after receiving the COVID-19 vaccine. In order to assess the effects of a daily consumption of 4 g plant stanols as their fatty acid esters in people with overweight, we recently began another double blind placebo-controlled intervention trial(Plat et al., 2022). - Clinical.Trials.gov: NCT04844346 |
| **33. Essential oil blend**  Participants receive an active essential oil blend to inhale for 15 minutes. The blend contains plant based oils sourced from flowers and citrus plants. | Mixed citrus  plants | Antimicrobial and against some food pathogenic microbes (Rhimi et al., 2022, Mohammed et al., 2022) | - **Essential** oils and their components were found to be mainly through inhibition of viral replication (El-gengaihi et al., 2020). - One of the main targets to reduce infection is ACE2, a receptor in host cells that facilitates virus cell entrance(Senthil Kumar et al., 2020). - It provided the initial proof that citronellol, geraniol, limonene, linalool, and neryl acetate, the main components of geranium and lemon essential oils, may inhibit ACE2 expression in epithelial cells, inhibiting virus entry into host cells and ultimately preventing viral infection. - Uncovering the underlying molecular processes of this inhibitory impact requires further research. | - The Effect of Aromatherapy on COVID-19-induced Anxiety   Clinical.Trials.gov: NCT04495842, NCT05114655 |

**References**

AHMAD, J., KHAN, I., BLUNDELL, R., AZZOPARDI, J. & MAHOMOODALLY, M. F. 2020. Stevia rebaudiana Bertoni.: An updated review of its health benefits, industrial applications and safety. *Trends in Food Science & Technology,* 100**,** 177-189.

AL-KURAISHY, H. M., AL-FAKHRANY, O. M., ELEKHNAWY, E., AL-GAREEB, A. I., ALORABI, M., DE WAARD, M., ALBOGAMI, S. M. & BATIHA, G. E.-S. 2022. Traditional herbs against COVID-19: back to old weapons to combat the new pandemic. *European Journal of Medical Research,* 27**,** 186.

AM LEE, S., LEE, S. H., KIM, J. Y. & LEE, W. S. 2019. Effects of glycyrrhizin on lipopolysaccharide-induced acute lung injury in a mouse model. *Journal of thoracic disease,* 11**,** 1287.

ARMANINI, D., FIORE, C., BIELENBERG, J., SABBADIN, C. & BORDIN, L. 2020. Coronavirus-19: possible therapeutic implications of spironolactone and dry extract of Glycyrrhiza glabra L.(Licorice). *Frontiers in Pharmacology,* 11.

ARREOLA, R., QUINTERO-FABIÁN, S., LÓPEZ-ROA, R. I., FLORES-GUTIÉRREZ, E. O., REYES-GRAJEDA, J. P., CARRERA-QUINTANAR, L. & ORTUÑO-SAHAGÚN, D. 2015. Immunomodulation and anti-inflammatory effects of garlic compounds. *Journal of immunology research,* 2015.

ATTIA, G. H., MARREZ, D. A., MOHAMMED, M. A., ALBARQI, H. A., IBRAHIM, A. M. & RAEY, M. A. E. 2021. Synergistic Effect of Mandarin Peels and Hesperidin with Sodium Nitrite against Some Food Pathogen Microbes. *Molecules,* 26**,** 3186.

BABAEI, F., NASSIRI‐ASL, M. & HOSSEINZADEH, H. 2020. Curcumin (a constituent of turmeric): New treatment option against COVID‐19. *Food science & nutrition,* 8**,** 5215-5227.

BACHIEGA, T. F., DE SOUSA, J. P. B., BASTOS, J. K. & SFORCIN, J. M. 2012. Clove and eugenol in noncytotoxic concentrations exert immunomodulatory/anti-inflammatory action on cytokine production by murine macrophages. *Journal of Pharmacy and Pharmacology,* 64**,** 610-616.

BAILLY, C. & VERGOTEN, G. 2020. Glycyrrhizin: An alternative drug for the treatment of COVID-19 infection and the associated respiratory syndrome? *Pharmacology & therapeutics,* 214**,** 107618.

BALKRISHNA, A., POKHREL, S., SINGH, J. & VARSHNEY, A. 2020. Withanone from Withania somnifera may inhibit novel coronavirus (COVID-19) entry by disrupting interactions between viral S-protein receptor binding domain and host ACE2 receptor.

BALKRISHNA, A., POKHREL, S. & VARSHNEY, A. 2021. Tinocordiside from Tinospora cordifolia (Giloy) may curb SARS-CoV-2 contagion by disrupting the electrostatic interactions between host ACE2 and viral S-protein receptor binding domain. *Combinatorial Chemistry & High Throughput Screening,* 24**,** 1795-1802.

BANERJEE, A., CZINN, S. J., REITER, R. J. & BLANCHARD, T. G. 2020. Crosstalk between endoplasmic reticulum stress and anti-viral activities: A novel therapeutic target for COVID-19. *Life sciences,* 255**,** 117842.

BANSAL, M., SINGH, N., PAL, S., DEV, I. & ANSARI, K. M. 2018. Chemopreventive role of dietary phytochemicals in colorectal cancer. *Advances in molecular toxicology,* 12**,** 69-121.

BARBOZA, J. N., DA SILVA MAIA BEZERRA FILHO, C., SILVA, R. O., MEDEIROS, J. V. R. & DE SOUSA, D. P. 2018. An overview on the anti-inflammatory potential and antioxidant profile of eugenol. *Oxidative medicine and cellular longevity,* 2018.

BORLINGHAUS, J., ALBRECHT, F., GRUHLKE, M. C., NWACHUKWU, I. D. & SLUSARENKO, A. J. 2014. Allicin: chemistry and biological properties. *Molecules,* 19**,** 12591-12618.

BUAGAEW, A. & POOMIPARK, N. 2020. Protective effect of piperine from Piper chaba fruits on LPS-induced inflammation in human intestinal cell line. *Journal of Medicinal Plants Research,* 14**,** 438-444.

CAO, Z., FANG, Y., LU, Y., TAN, D., DU, C., LI, Y., MA, Q., YU, J., CHEN, M. & ZHOU, C. 2017. Melatonin alleviates cadmium‐induced liver injury by inhibiting the TXNIP‐NLRP3 inflammasome. *Journal of Pineal Research,* 62**,** e12389.

CHEKALINA, N., BURMAK, Y., PETROV, Y., BORISOVA, Z., MANUSHA, Y., KAZAKOV, Y. & KAIDASHEV, I. 2018. Quercetin reduces the transcriptional activity of NF-kB in stable coronary artery disease. *Indian Heart Journal,* 70**,** 593-597.

CHEN, L.-X., HE, H. & QIU, F. 2011. Natural withanolides: an overview. *Natural product reports,* 28**,** 705-740.

CHOURASIA, M., KOPPULA, P. R., BATTU, A., OUSEPH, M. M. & SINGH, A. K. 2021. EGCG, a green tea catechin, as a potential therapeutic agent for symptomatic and asymptomatic SARS-CoV-2 infection. *Molecules,* 26**,** 1200.

CHOWDHURY, P. 2020. In silico investigation of phytoconstituents from Indian medicinal herb ‘Tinospora cordifolia (giloy)’against SARS-CoV-2 (COVID-19) by molecular dynamics approach. *Journal of Biomolecular Structure and Dynamics***,** 1-18.

CHUNG, H. Y. 2019. Anti-inflammatory and antioxidant activities of piperine on t. BHP-induced in Ac2F cells.

CIAMPI, E., URIBE-SAN-MARTIN, R., CÁRCAMO, C., CRUZ, J. P., REYES, A., REYES, D., PINTO, C., VÁSQUEZ, M., BURGOS, R. A. & HANCKE, J. 2020. Efficacy of andrographolide in not active progressive multiple sclerosis: a prospective exploratory double-blind, parallel-group, randomized, placebo-controlled trial. *BMC neurology,* 20**,** 1-10.

COLUNGA BIANCATELLI, R. M. L., BERRILL, M., CATRAVAS, J. D. & MARIK, P. E. 2020. Quercetin and vitamin C: an experimental, synergistic therapy for the prevention and treatment of SARS-CoV-2 related disease (COVID-19). *Frontiers in immunology,* 11**,** 1451.

CULPITT, S., ROGERS, D., FENWICK, P., SHAH, P., DE MATOS, C., RUSSELL, R., BARNES, P. & DONNELLY, L. 2003. Inhibition by red wine extract, resveratrol, of cytokine release by alveolar macrophages in COPD. *Thorax,* 58**,** 942-946.

DEFTEREOS, S. G., GIANNOPOULOS, G., VRACHATIS, D. A., SIASOS, G. D., GIOTAKI, S. G., GARGALIANOS, P., METALLIDIS, S., SIANOS, G., BALTAGIANNIS, S. & PANAGOPOULOS, P. 2020. Effect of colchicine vs standard care on cardiac and inflammatory biomarkers and clinical outcomes in patients hospitalized with coronavirus disease 2019: the GRECCO-19 randomized clinical trial. *JAMA network open,* 3**,** e2013136-e2013136.

DIBAZAR, S. P., FATEH, S. & DANESHMANDI, S. 2015. Immunomodulatory effects of clove (Syzygium aromaticum) constituents on macrophages: in vitro evaluations of aqueous and ethanolic components. *Journal of immunotoxicology,* 12**,** 124-131.

DINDA, A., GITMAN, M. & SINGHAL, P. C. 2005. Immunomodulatory effect of morphine: therapeutic implications. *Expert opinion on drug safety,* 4**,** 669-675.

DJALDETTI, M. & BESSLER, H. 2017. Nicotine modifies cytokine production by human mononuclears stimulated by colon cancer cells. *Colorectal Canc,* 3.

DORRI, M., HASHEMITABAR, S. & HOSSEINZADEH, H. 2018. Cinnamon (Cinnamomum zeylanicum) as an antidote or a protective agent against natural or chemical toxicities: a review. *Drug and chemical toxicology,* 41**,** 338-351.

DZOYEM, J., MCGAW, L., KUETE, V. & BAKOWSKY, U. 2017. Anti-inflammatory and anti-nociceptive activities of African medicinal spices and vegetables. *Medicinal spices and vegetables from Africa.* Elsevier.

EL-GENGAIHI, S. E., HAMED, M. A., KHALAF-ALLAH, A. E.-R. M. & MOHAMMED, M. A. 2013. Golden berry juice attenuates the severity of hepatorenal injury. *Journal of dietary supplements,* 10**,** 357-369.

EL-GENGAIHI, S. E., MOHAMMED, M. A., ABOUBAKER, D., SHOAIB, R. M., ASKER, M., ABDELHAMID, S. & HASSAN, E. 2020. Chemical, biological, and molecular studies on different citrus species wastes. *Plant Arch,* 20**,** 2773-2782.

EL-SABER BATIHA, G., MAGDY BESHBISHY, A., G WASEF, L., ELEWA, Y. H., A AL-SAGAN, A., EL-HACK, A., MOHAMED, E., TAHA, A. E., M ABD-ELHAKIM, Y. & PRASAD DEVKOTA, H. 2020. Chemical constituents and pharmacological activities of garlic (Allium sativum L.): A review. *Nutrients,* 12**,** 872.

ELFIKY, A. A. 2021. Natural products may interfere with SARS-CoV-2 attachment to the host cell. *Journal of Biomolecular Structure and Dynamics,* 39**,** 3194-3203.

ENMOZHI, S. K., RAJA, K., SEBASTINE, I. & JOSEPH, J. 2021. Andrographolide as a potential inhibitor of SARS-CoV-2 main protease: An in silico approach. *Journal of Biomolecular Structure and Dynamics,* 39**,** 3092-3098.

FEI, L., JIFENG, F., TIANTIAN, W., YI, H. & LINGHUI, P. 2017. Glycyrrhizin ameliorate ischemia reperfusion lung injury through downregulate TLR2 signaling cascade in alveolar macrophages. *Frontiers in pharmacology,* 8**,** 389.

FU, Q., CUI, Q., YANG, Y., ZHAO, X., SONG, X., WANG, G., BAI, L., CHEN, S., TIAN, Y. & ZOU, Y. 2018. Effect of resveratrol dry suspension on immune function of piglets. *Evidence-Based Complementary and Alternative Medicine,* 2018.

FUKADA, T., KATO, H., OZAKI, M. & YAGI, J. 2016. Impact of the timing of morphine administration on lipopolysaccharide-mediated lethal endotoxic shock in mice. *Shock: Injury, Inflammation, and Sepsis: Laboratory and Clinical Approaches,* 45**,** 564-569.

FUKADA, T., NAKAYAMA, R., IWAKIRI, H., KATO, H., YAGI, J. & OZAKI, M. 2012. Opioids inhibit lipopolysaccharide-mediated lethal shock in mice.

GAO, X., XU, Y. X., JANAKIRAMAN, N., CHAPMAN, R. A. & GAUTAM, S. C. 2001. Immunomodulatory activity of resveratrol: suppression of lymphocyte proliferation, development of cell-mediated cytotoxicity, and cytokine production. *Biochemical pharmacology,* 62**,** 1299-1308.

GAUTAM, S., GAUTAM, A., CHHETRI, S. & BHATTARAI, U. 2020. Immunity against COVID-19: potential role of Ayush Kwath. *Journal of Ayurveda and integrative medicine*.

GONZALEZ-RUBIO, J., NAVARRO-LOPEZ, C., LOPEZ-NAJERA, E., LOPEZ-NAJERA, A., JIMENEZ-DIAZ, L., NAVARRO-LOPEZ, J. D. & NAJERA, A. 2020. Cytokine release syndrome (CRS) and nicotine in COVID-19 patients: trying to calm the storm. *Frontiers in immunology,* 11**,** 1359.

GORGANI, L., MOHAMMADI, M., NAJAFPOUR, G. D. & NIKZAD, M. 2017. Piperine—the bioactive compound of black pepper: from isolation to medicinal formulations. *Comprehensive Reviews in Food Science and Food Safety,* 16**,** 124-140.

GUPTA, M. & KAUR, G. 2019. Withania somnifera (L.) Dunal ameliorates neurodegeneration and cognitive impairments associated with systemic inflammation. *BMC complementary and alternative medicine,* 19**,** 1-18.

HAMED, M. A., MOHAMMED, M. A., ABOUL NASER, A. F., MATLOUB, A. A., FAYED, D. B., ALI, S. A. & KHALIL, W. K. 2019. Optimization of curcuminoids extraction for evaluation against Parkinson’s disease in rats. *Journal of Biologically Active Products from Nature,* 9**,** 335-351.

HARIDAS, M., SASIDHAR, V., NATH, P., ABHITHAJ, J., SABU, A. & RAMMANOHAR, P. 2021. Compounds of Citrus medica and Zingiber officinale for COVID-19 inhibition: in silico evidence for cues from Ayurveda. *Future Journal of Pharmaceutical Sciences,* 7**,** 1-9.

HELLOU, E., MOHSIN, J., ELEMY, A., HAKIM, F., MUSTAFA‐HELLOU, M. & HAMOUD, S. 2022. Effect of ArtemiC in patients with COVID‐19: A Phase II prospective study. *Journal of Cellular and Molecular Medicine*.

HEWLINGS, S. J. & KALMAN, D. S. 2017. Curcumin: a review of its effects on human health. *Foods,* 6**,** 92.

HO, S.-C., CHANG, K.-S. & CHANG, P.-W. 2013. Inhibition of neuroinflammation by cinnamon and its main components. *Food chemistry,* 138**,** 2275-2282.

HUI, Q., AMMETER, E., LIU, S., YANG, R., LU, P., LAHAYE, L. & YANG, C. 2020. Eugenol attenuates inflammatory response and enhances barrier function during lipopolysaccharide-induced inflammation in the porcine intestinal epithelial cells. *Journal of Animal Science,* 98**,** skaa245.

JAYAKUMAR, T., HSIEH, C.-Y., LEE, J.-J. & SHEU, J.-R. 2013. Experimental and clinical pharmacology of Andrographis paniculata and its major bioactive phytoconstituent andrographolide. *Evidence-Based Complementary and Alternative Medicine,* 2013.

KANG, O.-H., CHOI, J.-G., LEE, J.-H. & KWON, D.-Y. 2010. Luteolin isolated from the flowers of Lonicera japonica suppresses inflammatory mediator release by blocking NF-κB and MAPKs activation pathways in HMC-1 cells. *Molecules,* 15**,** 385-398.

KAO, T.-K., OU, Y.-C., LIN, S.-Y., PAN, H.-C., SONG, P.-J., RAUNG, S.-L., LAI, C.-Y., LIAO, S.-L., LU, H.-C. & CHEN, C.-J. 2011. Luteolin inhibits cytokine expression in endotoxin/cytokine-stimulated microglia. *The Journal of nutritional biochemistry,* 22**,** 612-624.

KASHYAP, V. K., DHASMANA, A., YALLAPU, M. M., CHAUHAN, S. C. & JAGGI, M. 2020. Withania somnifera as a potential future drug molecule for COVID-19. Future Science.

KATO, K., YAMASHITA, S., KITANAKA, S. & TOYOSHIMA, S. 2001. Effect of gallic acid derivatives on secretion of Th1 cytokines and Th2 cytokines from anti CD3-stimulated spleen cells. *Yakugaku zasshi: Journal of the Pharmaceutical Society of Japan,* 121**,** 451-457.

KHALAF-ALLAH, A. E.-R. M., EL-GENGAIHI, S. E., HAMED, M. A., ZAHRAN, H. G. & MOHAMMED, M. A. 2016. Chemical composition of golden berry leaves against hepato-renal fibrosis. *Journal of dietary supplements,* 13**,** 378-392.

KHALIL, A. A. & RAHMAN, U. 2017. Ur; Khan, MR; Sahar, A.; Mehmood, T.; Khan, M. *Essential Oil Eugenol: Sources, Extraction Techniques and Nutraceutical Perspectives. RSC Adv,* 7**,** 32669-32681.

KHAN, F. R., KAZMI, S. M. R., IQBAL, N. T., IQBAL, J., ALI, S. T. & ABBAS, S. A. 2020. A quadruple blind, randomised controlled trial of gargling agents in reducing intraoral viral load among hospitalised COVID-19 patients: A structured summary of a study protocol for a randomised controlled trial. *Trials,* 21**,** 1-4.

KIM, G. D., LEE, S. E., KIM, T. H., JIN, Y. H., PARK, Y. S. & PARK, C. S. 2012. Melatonin suppresses acrolein‐induced IL‐8 production in human pulmonary fibroblasts. *Journal of pineal research,* 52**,** 356-364.

KIM, S.-H., JUN, C.-D., SUK, K., CHOI, B.-J., LIM, H., PARK, S., LEE, S. H., SHIN, H.-Y., KIM, D.-K. & SHIN, T.-Y. 2006. Gallic acid inhibits histamine release and pro-inflammatory cytokine production in mast cells. *Toxicological Sciences,* 91**,** 123-131.

KIM, S. H. & LEE, Y. C. 2009. Piperine inhibits eosinophil infiltration and airway hyperresponsiveness by suppressing T cell activity and Th2 cytokine production in the ovalbumin‐induced asthma model. *Journal of Pharmacy and Pharmacology,* 61**,** 353-359.

KONG, D., WANG, Z., TIAN, J., LIU, T. & ZHOU, H. 2019. Glycyrrhizin inactivates toll‐like receptor (TLR) signaling pathway to reduce lipopolysaccharide‐induced acute lung injury by inhibiting TLR2. *Journal of cellular physiology,* 234**,** 4597-4607.

KOSHAK, A. E., KOSHAK, E. A., MOBEIREEK, A. F., BADAWI, M. A., WALI, S. O., MALIBARY, H. M., ATWAH, A. F., ALHAMDAN, M. M., ALMALKI, R. A. & MADANI, T. A. 2021. Nigella sativa for the treatment of COVID-19: An open-label randomized controlled clinical trial. *Complementary therapies in medicine,* 61**,** 102769.

KUMAR, A., PRASAD, G., SRIVASTAV, S., GAUTAM, V. K. & SHARMA, N. 2020. Efficacy and safety of Guduchi Ghan vati in the management of asymptomatic COVID-19 infection: an open label feasibility study. *MedRxiv*.

KUROSE, K. & YATAGAI, M. 2005. Components of the essential oils of Azadirachta indica A. Juss, Azadirachta siamensis Velton, and Azadirachta excelsa (Jack) Jacobs and their comparison. *Journal of Wood Science,* 51**,** 185-188.

LANG, A., LAHAV, M., SAKHNINI, E., BARSHACK, I., FIDDER, H. H., AVIDAN, B., BARDAN, E., HERSHKOVIZ, R., BAR-MEIR, S. & CHOWERS, Y. 2004. Allicin inhibits spontaneous and TNF-α induced secretion of proinflammatory cytokines and chemokines from intestinal epithelial cells. *Clinical nutrition,* 23**,** 1199-1208.

LEE, W., LEE, S. Y., SON, Y.-J. & YUN, J.-M. 2015. Gallic acid decreases inflammatory cytokine secretion through histone acetyltransferase/histone deacetylase regulation in high glucose-induced human monocytes. *Journal of medicinal food,* 18**,** 793-801.

LI, Y., HE, S., TANG, J., DING, N., CHU, X., CHENG, L., DING, X., LIANG, T., FENG, S. & RAHMAN, S. U. 2017. Andrographolide inhibits inflammatory cytokines secretion in LPS-stimulated RAW264. 7 cells through suppression of NF-κB/MAPK signaling pathway. *Evidence-based Complementary and Alternative Medicine,* 2017.

LI, Y., YAO, J., HAN, C., YANG, J., CHAUDHRY, M., WANG, S., LIU, H. & YIN, Y. 2016. Quercetin, inflammation and immunity. Nutrients 8: 167.

LIANG, Y.-D., BAI, W.-J., LI, C.-G., XU, L.-H., WEI, H.-X., PAN, H., HE, X.-H. & OUYANG, D.-Y. 2016. Piperine suppresses pyroptosis and interleukin-1β release upon ATP triggering and bacterial infection. *Frontiers in pharmacology,* 7**,** 390.

LIN, Y., SHI, R., WANG, X. & SHEN, H.-M. 2008. Luteolin, a flavonoid with potential for cancer prevention and therapy. *Current cancer drug targets,* 8**,** 634-646.

LIU, J., BODNAR, B. H., MENG, F., KHAN, A. I., WANG, X., SARIBAS, S., WANG, T., LOHANI, S. C., WANG, P. & WEI, Z. 2021. Epigallocatechin gallate from green tea effectively blocks infection of SARS-CoV-2 and new variants by inhibiting spike binding to ACE2 receptor. *Cell & bioscience,* 11**,** 1-15.

LIU, Z. & YING, Y. 2020. The inhibitory effect of curcumin on virus-induced cytokine storm and its potential use in the associated severe pneumonia. *Frontiers in cell and developmental biology,* 8**,** 479.

LODHI, S., VADNERE, G. P., PATIL, K. D. & PATIL, T. P. 2020. Protective effects of luteolin on injury induced inflammation through reduction of tissue uric acid and pro-inflammatory cytokines in rats. *Journal of traditional and complementary medicine,* 10**,** 60-69.

LU, J., MA, Y., WU, J., HUANG, H., WANG, X., CHEN, Z., CHEN, J., HE, H. & HUANG, C. 2019. A review for the neuroprotective effects of andrographolide in the central nervous system. *Biomedicine & Pharmacotherapy,* 117**,** 109078.

MADERA-SALCEDO, I. K., CRUZ, S. L. & GONZALEZ-ESPINOSA, C. 2011. Morphine decreases early peritoneal innate immunity responses in Swiss–Webster and C57BL6/J mice through the inhibition of mast cell TNF-α release. *Journal of neuroimmunology,* 232**,** 101-107.

MALAGUARNERA, L. 2019. Influence of resveratrol on the immune response. *Nutrients,* 11**,** 946.

MANDLIK, D. S. & NAMDEO, A. G. 2021. Pharmacological evaluation of Ashwagandha highlighting its healthcare claims, safety, and toxicity aspects. *Journal of dietary supplements,* 18**,** 183-226.

MARINELLA, M. A. 2020. Indomethacin and resveratrol as potential treatment adjuncts for SARS‐CoV‐2/COVID‐19. *International journal of clinical practice,* 74**,** e13535.

MAURYA, S. P., DAS, B. K., SINGH, R. & TYAGI, S. 2019. Effect of Withania somnifer on CD38 expression on CD8+ T lymphocytes among patients of HIV infection. *Clinical Immunology,* 203**,** 122-124.

MAURYA, V. K., KUMAR, S., PRASAD, A. K., BHATT, M. L. & SAXENA, S. K. 2020. Structure-based drug designing for potential antiviral activity of selected natural products from Ayurveda against SARS-CoV-2 spike glycoprotein and its cellular receptor. *Virusdisease,* 31**,** 179-193.

MEHRBOD, P., ABDALLA, M. A., FOTOUHI, F., HEIDARZADEH, M., ARO, A. O., ELOFF, J. N., MCGAW, L. J. & FASINA, F. O. 2018. Immunomodulatory properties of quercetin-3-O-α-L-rhamnopyranoside from Rapanea melanophloeos against influenza a virus. *BMC complementary and alternative medicine,* 18**,** 1-10.

MENG, W., XIAOLIANG, R., XIUMEI, G., VINCIERI, F. F. & BILIA, A. R. 2009. Stability of active ingredients of traditional Chinese medicine (TCM). *Natural Product Communications,* 4**,** 1934578X0900401229.

MIN, K. J., JANG, J. H. & KWON, T. K. 2012. Inhibitory effects of melatonin on the lipopolysaccharide‐induced CC chemokine expression in BV2 murine microglial cells are mediated by suppression of Akt‐induced NF‐κB and STAT/GAS activity. *Journal of pineal research,* 52**,** 296-304.

MOHAMMED, M. A., ELGAMMAL, E. W., GAARA, A. H. & EL RAEY, M. A. 2022. Synergistic effect of Silver and ZnO nanoparticles green synthesized by Vitis vinifera stem extract with Ampicillin against some pathogenic microbes. *Egyptian Journal of Chemistry,* 65**,** 1-2.

MURCK, H. 2020. Symptomatic protective action of glycyrrhizin (licorice) in COVID-19 infection? *Frontiers in immunology,* 11**,** 1239.

NABAVI, S. F., SAHEBNASAGH, A., SHAHBAZI, A., NABAVI, S. M., AZIMI, S., KASHANI, M. K., HABTEMARIAM, S., RAHMANI, M., BADIEE, M. & HASHEMI, J. 2023. Putative Therapeutic Impact of Inflammasome Inhibitors Against COVID-19-Induced ARDS. *Current medicinal chemistry*.

NATIONAL ACADEMIES OF SCIENCES, E. & MEDICINE 2017. The health effects of cannabis and cannabinoids: the current state of evidence and recommendations for research.

NAWAZ, M. A., HUANG, Y., BIE, Z., AHMED, W., REITER, R. J., NIU, M. & HAMEED, S. 2016. Corrigendum: Melatonin: Current status and future perspectives in plant science. *Frontiers in plant science,* 7**,** 714.

NIE, X., CHEN, S.-R., WANG, K., PENG, Y., WANG, Y.-T., WANG, D., WANG, Y. & ZHOU, G.-C. 2017. Attenuation of innate immunity by andrographolide derivatives through NF-κB signaling pathway. *Scientific reports,* 7**,** 1-10.

ORTEGA-PEÑA, M. & GONZÁLEZ-CUEVAS, R. 2021. Familiar dermatologic drugs as therapies for COVID-19. *Actas Dermo-Sifiliográficas (English Edition),* 112**,** 118-126.

PANDIT, M. & LATHA, N. 2020. In silico studies reveal potential antiviral activity of phytochemicals from medicinal plants for the treatment of COVID-19 infection.

PETER, A. E., SANDEEP, B., RAO, B. G. & KALPANA, V. L. 2021. Calming the storm: natural Immunosuppressants as adjuvants to target the cytokine storm in COVID-19. *Frontiers in Pharmacology,* 11**,** 2305.

PIANTONI, S., PATRONI, A., TONIATI, P., FURLONI, R., FRANCESCHINI, F., ANDREOLI, L. & SCARSI, M. 2020. Why not to use colchicine in COVID-19? An oldanti-inflammatory drug for a novel auto-inflammatory disease. *Rheumatology,* 59**,** 1769-1770.

PIAO, W.-H., CAMPAGNOLO, D., DAYAO, C., LUKAS, R. J., WU, J. & SHI, F.-D. 2009. Nicotine and inflammatory neurological disorders. *Acta Pharmacologica Sinica,* 30**,** 715-722.

PLAT, J., VAN BRAKEL, L. & MENSINK, R. P. 2022. Plant stanol esters might optimise the immune response and improve the SARS-CoV-2/COVID-19 vaccine efficacy in overweight and obese subjects. *British Journal of Nutrition,* 127**,** 1117-1118.

PRAMOD, K., ANSARI, S. H. & ALI, J. 2010. Eugenol: a natural compound with versatile pharmacological actions. *Natural product communications,* 5**,** 1934578X1000501236.

PRASANTH, D., MURAHARI, M., CHANDRAMOHAN, V., PANDA, S. P., ATMAKURI, L. R. & GUNTUPALLI, C. 2020. In silico identification of potential inhibitors from Cinnamon against main protease and spike glycoprotein of SARS CoV-2. *Journal of Biomolecular Structure and Dynamics***,** 1-15.

RAHARDJO, B., WIDJAJANTO, E., SUJUTI, H. & KEMAN, K. 2014. Curcumin decreased level of proinflammatory cytokines in monocyte cultures exposed to preeclamptic plasma by affecting the transcription factors NF-κB and PPAR-γ. *Biomarkers and Genomic Medicine,* 6**,** 105-115.

REITER, R. J., SHARMA, R., MA, Q., DOMINQUEZ-RODRIGUEZ, A., MARIK, P. E. & ABREU-GONZALEZ, P. 2020. Melatonin inhibits COVID-19-induced cytokine storm by reversing aerobic glycolysis in immune cells: a mechanistic analysis. *Medicine in drug discovery,* 6**,** 100044.

RHIMI, W., MOHAMMED, M. A., ZAREA, A. A. K., GRECO, G., TEMPESTA, M., OTRANTO, D. & CAFARCHIA, C. 2022. Antifungal, antioxidant and antibiofilm activities of essential oils of Cymbopogon spp. *Antibiotics,* 11**,** 829.

RIBEIRO, D., FREITAS, M., TOMÉ, S. M., SILVA, A. M., LAUFER, S., LIMA, J. L. & FERNANDES, E. 2015. Flavonoids inhibit COX-1 and COX-2 enzymes and cytokine/chemokine production in human whole blood. *Inflammation,* 38**,** 858-870.

RIEDER, S. A., NAGARKATTI, P. & NAGARKATTI, M. 2012. Multiple anti‐inflammatory pathways triggered by resveratrol lead to amelioration of staphylococcal enterotoxin B‐induced lung injury. *British journal of pharmacology,* 167**,** 1244-1258.

SAGAR, V. & KUMAR, A. H. 2020. Efficacy of natural compounds from Tinospora cordifolia against SARS-CoV-2 protease, surface glycoprotein and RNA polymerase. *Virology***,** 1-10.

SAHA, P., KATARKAR, A., DAS, B., BHATTACHARYYA, A. & CHAUDHURI, K. 2016. 6-gingerol inhibits Vibrio cholerae-induced proinflammatory cytokines in intestinal epithelial cells via modulation of NF-κB. *Pharmaceutical biology,* 54**,** 1606-1615.

SAJJADI, S., MEHREGAN, I. & TAHERI, M. 2015. Essential oil composition of Hypericum triquetrifolium Turra growing wild in Iran. *Research in Pharmaceutical Sciences,* 10**,** 90.

SALEHI, B., SHAROPOV, F., FOKOU, P. V. T., KOBYLINSKA, A., JONGE, L. D., TADIO, K., SHARIFI-RAD, J., POSMYK, M. M., MARTORELL, M. & MARTINS, N. 2019. Melatonin in medicinal and food plants: Occurrence, bioavailability, and health potential for humans. *Cells,* 8**,** 681.

SANNEGOWDA, K., VENKATESHA, S. & MOUDGIL, K. 2015. Tinospora cordifolia inhibits autoimmune arthritis by regulating key immune mediators of inflammation and bone damage. *International journal of immunopathology and pharmacology,* 28**,** 521-531.

SASIDHARAN, S., SARKAR, N. & SAUDAGAR, P. 2022. Discovery of compounds inhibiting SARS-COV-2 multi-targets. *Journal of Biomolecular Structure and Dynamics***,** 1-16.

SCARSI, M., PIANTONI, S., COLOMBO, E., AIRÓ, P., RICHINI, D., MICLINI, M., BERTASI, V., BIANCHI, M., BOTTONE, D. & CIVELLI, P. 2020. Association between treatment with colchicine and improved survival in a single-centre cohort of adult hospitalised patients with COVID-19 pneumonia and acute respiratory distress syndrome. *Annals of the rheumatic diseases,* 79**,** 1286-1289.

SCHINK, A., NAUMOSKA, K., KITANOVSKI, Z., KAMPF, C. J., FRÖHLICH-NOWOISKY, J., THINES, E., PÖSCHL, U., SCHUPPAN, D. & LUCAS, K. 2018. Anti-inflammatory effects of cinnamon extract and identification of active compounds influencing the TLR2 and TLR4 signaling pathways. *Food & function,* 9**,** 5950-5964.

SEIGNEURIC, C., CAMARA, B., DELMONT, J., BUSATO, F., PAYEN, J., ARMENGAUD, M. & MARCHOU, B. 2008. Quinquina and man. *Medecine Tropicale: Revue du Corps de Sante Colonial,* 68**,** 459-462.

SENTHIL KUMAR, K., GOKILA VANI, M., WANG, C.-S., CHEN, C.-C., CHEN, Y.-C., LU, L.-P., HUANG, C.-H., LAI, C.-S. & WANG, S.-Y. 2020. Geranium and lemon essential oils and their active compounds downregulate angiotensin-converting enzyme 2 (ACE2), a SARS-CoV-2 spike receptor-binding domain, in epithelial cells. *Plants,* 9**,** 770.

SHARIFI-RAD, J., CRISTINA CIRONE SILVA, N., JANTWAL, A., D BHATT, I., SHAROPOV, F., C CHO, W., TAHERI, Y. & MARTINS, N. 2019. Therapeutic potential of allicin-rich garlic preparations: emphasis on clinical evidence toward upcoming drugs formulation. *Applied Sciences,* 9**,** 5555.

SHARIFI-RAD, M., VARONI, E. M., SALEHI, B., SHARIFI-RAD, J., MATTHEWS, K. R., AYATOLLAHI, S. A., KOBARFARD, F., IBRAHIM, S. A., MNAYER, D. & ZAKARIA, Z. A. 2017. Plants of the genus Zingiber as a source of bioactive phytochemicals: From tradition to pharmacy. *Molecules,* 22**,** 2145.

SHARMA, U., BALA, M., KUMAR, N., SINGH, B., MUNSHI, R. K. & BHALERAO, S. 2012. Immunomodulatory active compounds from Tinospora cordifolia. *Journal of ethnopharmacology,* 141**,** 918-926.

SHARMA, V., KAUSHIK, S., PANDIT, P., DHULL, D., YADAV, J. P. & KAUSHIK, S. 2019. Green synthesis of silver nanoparticles from medicinal plants and evaluation of their antiviral potential against chikungunya virus. *Applied microbiology and biotechnology,* 103**,** 881-891.

SHENG, Y., WU, T., DAI, Y., JI, K., ZHONG, Y. & XUE, Y. 2020. The effect of 6-gingerol on inflammatory response and Th17/Treg balance in DSS-induced ulcerative colitis mice. *Annals of translational medicine,* 8.

SHIMIZU, K., FUNAMOTO, M., SUNAGAWA, Y., SHIMIZU, S., KATANASAKA, Y., MIYAZAKI, Y., WADA, H., HASEGAWA, K. & MORIMOTO, T. 2019. Anti-inflammatory action of curcumin and its use in the treatment of lifestyle-related diseases. *European Cardiology Review,* 14**,** 117.

SHREE, P., MISHRA, P., SELVARAJ, C., SINGH, S. K., CHAUBE, R., GARG, N. & TRIPATHI, Y. B. 2020. Targeting COVID-19 (SARS-CoV-2) main protease through active phytochemicals of ayurvedic medicinal plants–Withania somnifera (Ashwagandha), Tinospora cordifolia (Giloy) and Ocimum sanctum (Tulsi)–a molecular docking study. *Journal of Biomolecular Structure and Dynamics***,** 1-14.

SINHA, S. K., PRASAD, S. K., ISLAM, M. A., GURAV, S. S., PATIL, R. B., ALFARIS, N. A., ALDAYEL, T. S., ALKEHAYEZ, N. M., WABAIDUR, S. M. & SHAKYA, A. 2020. Identification of bioactive compounds from Glycyrrhiza glabra as possible inhibitor of SARS-CoV-2 spike glycoprotein and non-structural protein-15: a pharmacoinformatics study. *Journal of Biomolecular Structure and Dynamics***,** 1-15.

SORDILLO, P. P. & HELSON, L. 2015. Curcumin suppression of cytokine release and cytokine storm. A potential therapy for patients with Ebola and other severe viral infections. *in vivo,* 29**,** 1-4.

SRIVASTAVA, V., YADAV, A. & SARKAR, P. 2020. Molecular docking and ADMET study of bioactive compounds of Glycyrrhiza glabra against main protease of SARS-CoV2. *Materials Today: Proceedings*.

SUN, Y., JIANG, M., PARK, P.-H. & SONG, K. 2020. Transcriptional suppression of androgen receptor by 18β-glycyrrhetinic acid in LNCaP human prostate cancer cells. *Archives of pharmacal research,* 43**,** 433-448.

TANG, J., DIAO, P., SHU, X., LI, L. & XIONG, L. 2019. Quercetin and quercitrin attenuates the inflammatory response and oxidative stress in LPS-induced RAW264. 7 cells: in vitro assessment and a theoretical model. *BioMed research international,* 2019.

THAKAR, A., PANARA, K., SHAH, H., KALSARIYA, B., RUPAREL, S., JAIN, N., BHATT, P., JANI, D., DODIA, R. & PATEL, F. 2022. duduchi Ghanavati (Ayurveda medication) improves the perceived immunity in individuals at risk of SARS-CoV-2: A Multicentric, controlled, before-and-after study. *European Journal of Integrative Medicine***,** 102131.

THEOHARIDES, T. C. 2020. COVID‐19, pulmonary mast cells, cytokine storms, and beneficial actions of luteolin. *Biofactors (Oxford, England)*.

TRIPATHI, M. K., SINGH, P., SHARMA, S., SINGH, T. P., ETHAYATHULLA, A. & KAUR, P. 2021. Identification of bioactive molecule from Withania somnifera (Ashwagandha) as SARS-CoV-2 main protease inhibitor. *Journal of Biomolecular Structure and Dynamics,* 39**,** 5668-5681.

TRIPATHI, S., MAIER, K. G., BRUCH, D. & KITTUR, D. S. 2007. Effect of 6-gingerol on pro-inflammatory cytokine production and costimulatory molecule expression in murine peritoneal macrophages. *Journal of Surgical Research,* 138**,** 209-213.

UPADHYAY, A. K., KUMAR, K., KUMAR, A. & MISHRA, H. S. 2010. Tinospora cordifolia (Willd.) Hook. f. and Thoms.(Guduchi)–validation of the Ayurvedic pharmacology through experimental and clinical studies. *International journal of Ayurveda research,* 1**,** 112.

VALIZADEH, H., ABDOLMOHAMMADI-VAHID, S., DANSHINA, S., GENCER, M. Z., AMMARI, A., SADEGHI, A., ROSHANGAR, L., ASLANI, S., ESMAEILZADEH, A. & GHAEBI, M. 2020. Nano-curcumin therapy, a promising method in modulating inflammatory cytokines in COVID-19 patients. *International immunopharmacology,* 89**,** 107088.

VAN DE SAND, L., BORMANN, M., ALT, M., SCHIPPER, L., HEILINGLOH, C. S., TODT, D., DITTMER, U., ELSNER, C., WITZKE, O. & KRAWCZYK, A. 2020. Glycyrrhizin effectively neutralizes SARS-CoV-2 in vitro by inhibiting the viral main protease. *BioRxiv*.

VARMA, A., PADH, H. & SHRIVASTAVA, N. 2011. Andrographolide: a new plant-derived antineoplastic entity on horizon. *Evidence-Based Complementary and Alternative Medicine,* 2011.

WANG, W., WANG, J., DONG, S.-F., LIU, C.-H., ITALIANI, P., SUN, S.-H., XU, J., BORASCHI, D., MA, S.-P. & QU, D. 2010. Immunomodulatory activity of andrographolide on macrophage activation and specific antibody response. *Acta Pharmacologica Sinica,* 31**,** 191-201.

WONG, S. Y., TAN, M. G., BANKS, W. A., WONG, W. F., WONG, P. T.-H. & LAI, M. K. 2016. Andrographolide attenuates LPS-stimulated up-regulation of CC and CXC motif chemokines in rodent cortex and primary astrocytes. *Journal of neuroinflammation,* 13**,** 1-11.

YADAV, R., JEE, B. & AWASTHI, S. K. 2015. Curcumin suppresses the production of pro-inflammatory cytokine interleukin-18 in lipopolysaccharide stimulated murine macrophage-like cells. *Indian Journal of Clinical Biochemistry,* 30**,** 109-112.

ZAHEDIPOUR, F., HOSSEINI, S. A., SATHYAPALAN, T., MAJEED, M., JAMIALAHMADI, T., AL‐RASADI, K., BANACH, M. & SAHEBKAR, A. 2020. Potential effects of curcumin in the treatment of COVID‐19 infection. *Phytotherapy Research,* 34**,** 2911-2920.

ZHAI, W.-J., ZHANG, Z.-B., XU, N.-N., GUO, Y.-F., QIU, C., LI, C.-Y., DENG, G.-Z. & GUO, M.-Y. 2016. Piperine plays an anti-inflammatory role in Staphylococcus aureus endometritis by inhibiting activation of NF-κB and MAPK pathways in mice. *Evidence-Based Complementary and Alternative Medicine,* 2016.

ZHANG, L., LI, Y., GU, Z., WANG, Y., SHI, M., JI, Y., SUN, J., XU, X., ZHANG, L. & JIANG, J. 2015. Resveratrol inhibits enterovirus 71 replication and pro-inflammatory cytokine secretion in rhabdosarcoma cells through blocking IKKs/NF-κB signaling pathway. *PloS one,* 10**,** e0116879.

ZHANG, M., PAN, H., XU, Y., WANG, X., QIU, Z. & JIANG, L. 2017. Allicin decreases lipopolysaccharide-induced oxidative stress and inflammation in human umbilical vein endothelial cells through suppression of mitochondrial dysfunction and activation of Nrf2. *Cellular Physiology and Biochemistry,* 41**,** 2255-2267.

ZHANG, R., WANG, X., NI, L., DI, X., MA, B., NIU, S., LIU, C. & REITER, R. J. 2020. COVID-19: Melatonin as a potential adjuvant treatment. *Life sciences,* 250**,** 117583.

ZHAO, H., ZHAO, M., WANG, Y., LI, F. & ZHANG, Z. 2016. Glycyrrhizic acid prevents sepsis-induced acute lung injury and mortality in rats. *Journal of Histochemistry & Cytochemistry,* 64**,** 125-137.

ZHOU, Z. X., MOU, S. F., CHEN, X. Q., GONG, L. L. & GE, W. S. 2018. Anti-inflammatory activity of resveratrol prevents inflammation by inhibiting NF‑κB in animal models of acute pharyngitis. *Molecular medicine reports,* 17**,** 1269-1274.

ZHU, L., GU, P. & SHEN, H. 2019. Gallic acid improved inflammation via NF-κB pathway in TNBS-induced ulcerative colitis. *International immunopharmacology,* 67**,** 129-137.
